# Liver phosphorus content and liver function in states of phosphorus deficiency in transition dairy cows

**DOI:** 10.1371/journal.pone.0219546

**Published:** 2019-07-22

**Authors:** Walter Grünberg, Stefanie Witte, Imke Cohrs, Lennart Golbeck, Jos F. Brouwers, Anja E. Müller, M. Schmicke

**Affiliations:** 1 Clinic for Cattle, University of Veterinary Medicine Hannover, Foundation, Hanover, Germany; 2 Dept. Farm Animal Health, Utrecht University, CL Utrecht, The Netherlands; 3 Dept. Biochemistry and Cell Biology, CM Utrecht, The Netherlands; 4 IDEXX Laboratories, Ludwigsburg, Germany; National Veterinary School of Toulouse, FRANCE

## Abstract

Phosphorus (P) deficiency in early lactating dairy cows is receiving increased attention because of incentives aiming at curtailing environmental pollution with P by reducing dietary P in ruminant diets. An in-vitro study using bovine hepatocytes incubated for 7 days with phosphate (Pi) concentrations of 0.9, 1.8 or 2.7 mmol/L, and an in-vivo study feeding late pregnant dairy cows diets with either adequate (0.28% and 0.44% in DM ante-partum and post-partum respectively) or low P content (0.15% and 0.20% in DM ante-partum and post-partum respectively) from 4 weeks before to 4 weeks after calving were conducted to explore effects of P deprivation on liver function. In vitro the relative abundance of mRNA of key enzymes of the carbohydrate metabolism in incubated hepatocytes and liver metabolites in culture medium were determined. In vivo health and productivity of experimental cows on low and adequate dietary P supply were monitored, and liver tissue and blood samples were obtained repeatedly. Liver tissue was assayed for its triacylglycerol-, mineral and water content as well as for the relative abundance of mRNA of enzymes of the carbohydrate-, fat- and protein metabolism. Reduced Pi-availability was not associated with altered enzyme transcription rates or metabolic activity in-vitro. The most prominent clinical finding associated with P deprivation in-vivo was feed intake depression developing after the first week of lactation. Accordingly cows on low P diets had lower milk yield and showed more pronounced increases in liver triacylglycerol after calving. Although the liver P content decreased in P deficient cows, neither negative effects on enzyme transcription rates nor on blood parameters indicative of impaired liver metabolic activity or liver injury were identified. These results indicate the P deprivation only indirectly affects the liver through exacerbation of the negative energy balance occurring as P deficient cows become anorectic.

## Introduction

Postparturient hypophosphatemia, that is defined as subnormal plasma inorganic phosphorus concentration ([Pi]) occurring in the first days to weeks of lactation is commonly observed in healthy but also in sick dairy cows in early lactation [1–3]. The clinical relevance of this electrolyte imbalance remains uncertain and has been controversially debated for many years. Empirical evidence suggests that hypophosphatemia or phosphorus (P) deprivation in fresh cows may cause or at least contribute to conditions such as the downer cow syndrome or postparturient hemoglobinuria in dairy cows [4, 5]. While concerns of producers and veterinarians with P deficiency in periparturient cows focus on the conditions mentioned above, P deficiency and hypophosphatemia have been associated with a variety of other clinical signs and conditions in other species. The most consistently reported symptom of transient but severe hypophosphatemia across species seems to be the development of anorexia in affected individuals [6–10]. Other disturbances recognized in non-ruminant species but barely addressed in cattle include immune cell function disorders, cardiovascular disturbances as well as neurologic and liver function disorders [11–15]. In human medicine hypophosphatemia is well-recognized as potentially life threatening complication in patients with acute liver failure or after major liver resection. It is thought to be a sign of strong regenerative activity of the liver that depends on abundant availability of P for synthetic and metabolic activity of a rapidly growing number of hepatocytes [14, 16]. Hypophosphatemia and P deprivation were found to impair the regenerative activity of the liver in human patients with liver disease. Oral or parenteral P supplementation in contrast, as part of the conservative treatment of acute liver failure reduced the complication rate and improved the recovery rate in human patients [17]. An association of hypophosphatemia with disturbed liver function or liver injury as recognized in human medicine would be of relevance in bovine production medicine where liver function disorders caused by hepatic lipidosis are common in highly productive dairy cows in early lactation and occur concomitantly with hypophosphatemia [18, 19]. Associations between hypophosphatemia and blood biochemical parameters indicative of disturbed liver function or liver injury in early lactating dairy cattle have been reported in the past [1]. However because fresh cows with hepatic lipidosis often also suffer from concomitant disorders affecting dry matter intake a direct causative association between impaired liver function or liver injury and hypophosphatemia is difficult to establish.

With the increased awareness that excessive amounts of P in manure of production animals present an environmental concern, regulations aiming at reducing the fecal P output through a more restrictive use of P in ruminant rations are currently implemented in many parts of the world. Producers and veterinarians however have raised concerns that these incentives may present a risk for health and productivity of highly productive dairy cows particularly in early lactation. A reduction of the dietary P supply to dairy cows with continuously increasing milk production and thus continuously increasing loss of P through the mammary gland is thought to exacerbate severity and duration of postparturient hypophosphatemia [19]. There is a paucity of knowledge over the effects of hypophosphatemia and P deprivation particularly in transition dairy cows, making it difficult to assess in how far these concerns are scientifically justified.

The study presented here is part of a multi-institutional project investigating the effects of dietary P-deprivation in transition dairy cows on a variety of organs and tissues. The approach chosen for this purpose was to experimentally feed dairy cows either a markedly P deficient diet or a ration with adequate P content throughout the transition period and to compare effects of both treatments on a variety of organs and tissues, as well as on the individual cow as a whole. The dietary P content of the P deficient ration used in this project was the lowest level achievable when using standard dairy ration ingredients available in Europe. The level of dietary P deprivation that was achieved here is unlikely to be encountered in the field in Europe or the Americas. It can thus be assumed that more pronounced signs and symptoms of dietary P deprivation than what has been reported in the different studies of this multi-institutional project are unlikely to be observed under field conditions. The studies presented here aimed at investigating the effect of P deprivation on liver tissue and liver function in transition cows. Other laboratories have explored the effects of restricted dietary P supply to dairy cows in the transition period on immune cell function, muscle tissue composition and function as well as on the periparturient calcium balance [20–22]. In order to better be able to differentiate between direct and indirect effects of P deprivation on organ and cell function an in-vitro study conducted on bovine hepatocyte cell culture as well as an in-vivo study conducted in transition dairy cows were performed. We hypothesized that P deprivation of the liver would hamper the hepatic carbohydrate and other metabolic activities by limiting the availability of intracellular P required for many phosphorylation- and other P dependent biochemical reactions.

## Material and methods

### Ethics statement

Liver tissue required for the in-vitro study (part 1) was obtained from cows sacrificed for an unrelated project (permit no. 33.12-42502-04-15/2024, State Office for Consumer Protection and Food Safety, Lower Saxony, Germany). The clinical study (part 2) was approved by the Utrecht University Institutional Animal Care and Use Committee (permit no AVD108002016616) and was carried out in strict accordance with the national and institutional guidelines for the care and use of experimental animals.

### In-vitro study (part 1)

Primary bovine hepatocytes (PBH) were collected from liver tissue obtained immediately after slaughter from healthy cows.

#### Hepatocyte isolation and incubation

The caudate lobe of the liver was collected, immediately rinsed with 250 mL of ice-cold EGTA-containing buffer and 200 mL of EGTA-free buffer and kept on ice for the transport to the laboratory. The liver was then placed in 150 mL EGTA-free buffer at 37°C and briefly rinsed to facilitate identification of four blood vessels suitable for the following perfusion. A buttoned cannula was inserted into each of these vessels and attached with tissue glue (Histoacryl, Braun Surgical S.a., Rubi, Spain). The prepared lobe was then placed on a Buchner funnel, and a two-step perfusion with a perfusion rate of 40 mL/min using a peristaltic pump was initiated (Behr Labortechnik GmbH, Düsseldorf, Germany). The EGTA-containing buffer was saturated with carbogen (95% O_2_/5% CO_2_) 30 min prior to the perfusion; all perfusion buffers were at 37°C when reaching the liver. First, the liver was perfused with 200 mL of EGTA-containing buffer, followed by 200 mL of a calcium-containing buffer in a non-circulating system. Finally, 54 U collagenase P (36 mg dissolved in 100 mL calcium-containing buffer, Roche, Indianapolis, IN, USA) was pumped through the liver lobe using a re-circulating system. The collagenase perfusion was stopped once the tissue lost its structure. The time required for this process was 8 min. After the two-step perfusion, the liver lobe was transferred into a sterile glass petri dish, and the glissonian capsule was incised with a sterile scalpel. Hepatocytes were collected into 100 mL ice-cold Williams’ Medium E containing 20% fetal bovine serum (FBS, PAN BioTech, Aidenbach, Germany). The cell solution was filtered through gauze, centrifuged (60xg for 3 min at 4°C), and washed with ice-cold Williams’ Medium E with 10% FBS. The obtained cell pellet was then suspended in Williams’ Medium E (PAN BioTech, Aidenbach, Germany) and centrifuged again in order to wash the cells. Cells were purified using Easycoll (Merck, Darmstadt, Germany). Harvested hepatocytes were counted and viability was determined by a trypan blue exclusion test. The cells were seeded on twelve-well plates previously coated with rat tail collagen (1 mg/mL Collagen, Roche, Roche, Indianapolis, IN, USA) at a density of 5 x 10^5^ cells/well (1.37 cells/cm^2^) in 1.5 mL culture medium based on Williams’ Medium E with 10% FBS (S1A and S1B Table). After 3h of seeding, the medium was removed, and a second layer of rat tail collagen was added to perform the sandwich culture. Twenty minutes after polymerization of the second collagen layer 1 mL of Williams’ culture medium E without FBS or bovine insulin was added. Cells were cultured in humidified air with 5% CO_2_ at 37°C. After 15h of incubation in serum-free medium the culture medium was replaced by experimental culture media with different concentrations of inorganic phosphorus (Pi) and glucose (Table 1). These media contained no insulin but 1.25 mmol/L propionate, 1.0 mmol/L pyruvate and 1% non-essential amino acids were added. Three different Pi concentrations ([Pi]) in culture medium were used that were a low Pi medium (**LPi**) with 0.9 mmol/L, an intermediate [Pi] (**IPi**) with 1.8 mmol/L and high Pi (**HPi**) with 2.7 mmol/L. The media with the different [Pi] all contained 5 mmol/L glucose (Table 1). Two more experimental media, one without glucose and 1.8 mmol/L Pi (**0G**) and one with a high glucose concentration of 10 mmol/L and 1.8 mmol/L Pi (**group HG**) were prepared (Table 1). The concentrations of Pi ([Pi]) used in this study were arbitrarily chosen with the objective to mimic states of hypo-, normo- and hyperphosphatemia normally encountered in vivo in dairy cows [19].

**Table 1:** Concentrations of inorganic phosphorus ([Pi]) and glucose in the culture media used incubation of primary bovine hepatocytes of the different groups.

Experimental incubation media	[Pi] (mmol/L)	Glucose (mmol/L)
**0G**	0.9	0
**HG**	0.9	10
**LPi**	0.9	5
**IPi**	1.8	5
**HPi**	2.7	5

Experimental in-vitro treatments were 0G (zero glucose), HG (high glucose), LPi (low phosphorus), IPi (intermediated phosphorus) and HPi (high phosphorus)

Culture media were replaced after 6h of incubation for the first time (Time point 0h), again 24h after the start of incubation and then in 24h intervals. Each of the five experimental media was used to incubate 24 wells. For each of the 5 experimental media hepatocytes of 12 wells were harvested after 6h and after 72h of incubation. At collection the content of two wells with the same medium was pooled so that 6 cell samples were available at each sampling for each of the 5 media. Samples were briefly centrifuged, the supernatant removed and the cell pellets stored at -80°C until analysis.

#### Biochemical analysis of harvested experimental culture media

Experimental culture media collected after 6h (time point 0), 48h, 72h, 96h, 120h and 168h of incubation were assayed for the activity of lactate dehydrogenase (LDH, Lactate Dehydrogenase Activity Kit, Sigma-Aldrich. St. Louis, USA) as well as the concentrations of urea (Urea Assay Kit, Abcam, Cambridge, UK) and glucose (Glucose HK CP Kit, Horiba ABC, Cedex, France). Results from medium sampled after 24h are omitted as they only contained cell metabolites of 18h rather than 24h of incubation and thus were difficult to compare to the later time points.

For each sampling time the glucose balance was calculated by subtracting the glucose concentration in fresh medium from the glucose concentration measured in the sample. Positive values thus indicate net glucose synthesis while negative values indicate a net uptake of glucose by the incubated hepatocytes.

#### Analyses of mRNA expression

The metabolic activity of liver cells harvested after 6h and 72h of incubation in experimental medium was studied by measuring the relative abundance of mRNA of enzymes of the gluconeogenic pathway such as the cytosolic phosphoenolpyruvate carboxykinase (cPEPCK) and pyruvate carboxylase (PC), enzymes of the glycolytic pathway such as liver pyruvate kinase (PKL) and liver phosphofructokinase (PFKL) as well as mRNA of the gene encoding albumin (Alb). Further to this the nuclear factor kappaBlα (NFkBIα), a marker for programmed cell death and the liver specific transcription factor hepatocyte nuclear factor 4α (HNF4α) were studied using RT-qPCR. Total RNA extraction was initially performed using TRIzol Reagent (Sigma-Aldrich, St. Louis, USA). To assess the extracted RNA quality and quantity, relative integrity was determined using the RNA 6000 Nano Assay Kit and Agilent 2100 Bioanalyzer (Agilent Technologies, Böblingen, Germany). To transcribe RNA into cDNA, the BioRad Real Time System CFX96 1000 Touch (BioRad, Munich, Germany) was used according to the manufacturer’s instructions and as previously described [23]. A PCR reaction mix containing 25 ng/μL cDNA, 4 μL of HOT FIREPol EVA Green qPCR Supermix (5x) (Solis BioDyne, Tartu, Estonia), and 0.2 μM of each primer (Eurofins MWG Operon, Ebersberg, Germany) for the genes of interest was used. The primers were either previously described or constructed based on information of the public database https://www.ncbi.nlm.nih.gov/tools/primer-blast/index.cgi (S2 Table). The PCR cycler was programmed as follows: RNA denaturation at 95°C for 12 min followed by 40 cycles of 95°C for 15 s, 60°C for 30 s and 72°C for 30 s for amplification. EVA Green Dye (Biotium, Hayward, CA, USA) was used to visualize the fragments. A melting curve was generated to verify PCR fragments. This was initiated at 55°C, increasing to a final temperature of 95°C, with a temperature increase of 0.5°C every 10 s. Finally, the relative abundance of cPEPCK, PC, PKL, PFKL, HNF4α, NFκBIα and Alb in the cell samples relative to a calibrator was calculated using the ΔCt method with regard to the different efficiencies of the primers. For the calibrator cells were collected immediately after the purification step with Easycoll (Merck, Darmstadt, Germany). GAPDH was used as the reference gene.

### In-vivo study (part 2)

#### Animals and housing

A total of 36 healthy, multiparous, pregnant Holstein-Friesian or Holstein-Friesian cross breed dairy cows in late gestation were used in this study. Cows were purchased from commercial dairy farms in the Netherlands at least 1 month before enrollment. The study was conducted in two immediately consecutive replicates of 18 cows each. Cows of each replicate were bred within a period of 5 days in order to narrow the calving period of each replicate as much as possible. Cows were healthy as determined by physical, hematological and blood biochemical examination at the time of enrollment. After completion of the study cows remained at the research facility for use in further research or for undergraduate veterinary education.

Animals were housed in individual tie stalls with rubber bedding, covered with sawdust, in a temperature controlled facility. Study animals were dry for at least 2 weeks prior to enrollment.

#### Study design and experimental rations

**Fig 1:**
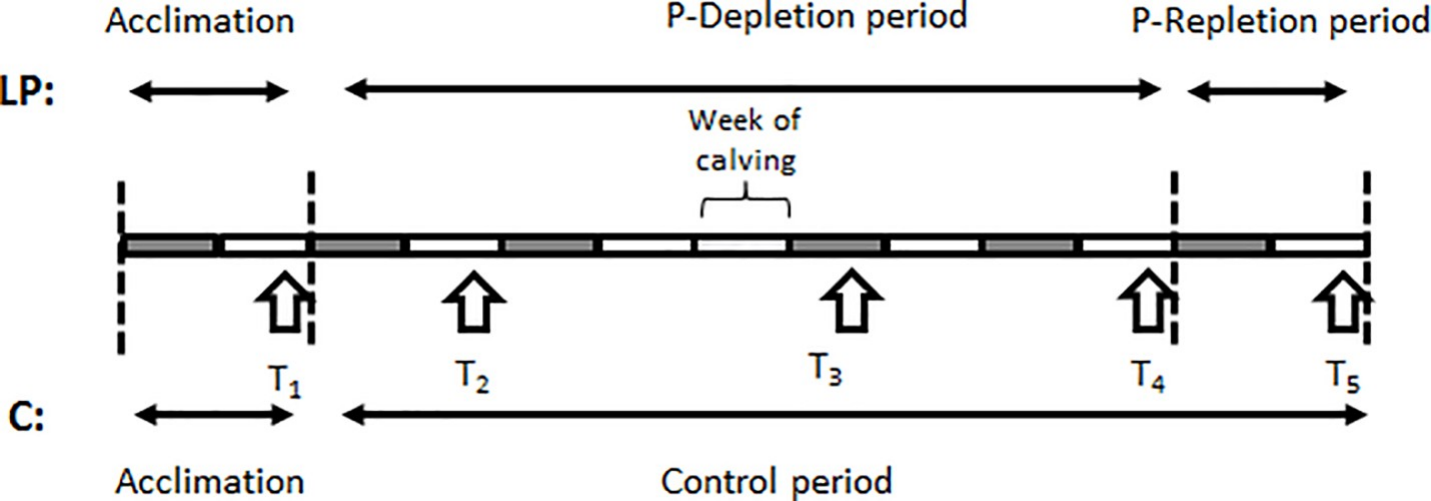
Experimental timeline of the in-vivo study (part 2). Each rectangle represents one week of time. Arrows represent the sampling times T_1_, T_2_, T_3_, T_4_, and T_5_ at which liver tissue specimens were obtained. Vertical dashed lines depict the beginning and end of each study phase for animals on low (LP) and adequate (C) phosphorus diet. During acclimation LP and C cows were offered the same dry cow ration with adequate phosphorus supply. Only cows of replicate 1 underwent the repletion period at the end of the study during which LP cows were switched to the lactation cow ration of C with adequate phosphorus content.

The study was conceived as replicated randomized controlled trial. Within each of the two replicates cows were paired by lactation number and 305 days milk yield of the previous lactation. Cows of each pair were then randomly distributed to the two treatments of this study that were low phosphorus (LP) and control (C). The study covered a minimum period of 11 weeks extending from 6 weeks before the expected week of calving to 4 weeks after the expected week of calving (Fig 1). The study commenced with a 2-weeks acclimation period during which cows of both treatments were fed identical rations with adequate P content. Thereafter cows assigned to LP were switched to a P-deficient diet from 4 weeks before the expected week of calving until 4 weeks after the expected week of calving, while cows assigned to C remained on a diet with adequate P supply (Fig 1). Cows of the first replicate underwent an additional 2-weeks P repletion period, immediately following the P-deprivation period during which both treatments again received the same ration for lactating cows with a P content slightly exceeding requirements [24] (Fig 1).

A diet based on corn silage, grass seed straw, beet pulp and soybean meal was offered as a total mixed ration (TMR) for dry cows and lactating cows depending on the stage of the lactation cycle. The same base ration formulated to meet or slightly exceed current recommendations for dairy cows, with exception of the dietary P content was prepared for both treatments [24] (S3 Table). Cows assigned to LP were offered this P-deficient base ration, while animals on treatment C received the base ration that was supplemented with NaH_2_PO_4_ to obtain a dietary P content meeting or slightly exceeding current recommendations for dairy cows [24]. The P contents of the experimental rations were 0.15% and 0.28% in dry matter (DM) for LP and C respectively during the dry period and 0.20% and 0.44% P in DM respectively during lactation (S3 Table). Feed was offered twice daily between 0600 and 0700 and between 1800 and 1900. Access to feed was restricted to 12.5 kg DM per day for dry cows but was ad libitum after calving. Cows had free access to water. Lactating cows were milked twice daily between 0600 and 0700 and between 1800 and 1900.

#### Sample collection and experimental procedures

Animal health, feed intake, milk production, and body mass. Attitude and behavior of the study animals were monitored daily, and a complete physical examination was conducted once a week. Cows were weighed on an electronic scale at the end of the acclimation period (**T**_**1**_), after 2 weeks of dietary P deprivation (**T**_**2**_), during the first week of lactation (**T**_**3**_), at the end of the depletion period (**T**_**4**_) and for cows of replicate 1 at the end of the repletion phase (**T**_**5**_); (Fig 1).

Milk yield and dry matter intake were recorded daily and the average daily dry matter feed intake (DMI) and milk yield of the 7 days prior to each of the five sampling times were calculated for every cow.

Urine was obtained at least every other day from every cow from the day of calving and grossly checked for discoloration suggestive of hemoglobinuria. The concentration of urine acetone and acetoacetic acid was determined semi-quantitatively with commercial urine sticks (Medi-Test Keton, Macherey-Nagel, Hoerdt, France). Animals with acetoacetic acid concentrations above 10 mmol/L but without concomitant clinical signs were considered to be subclinically ketotic and were treated orally with 250 mL propylene glycol twice daily, administered by drench gun until ketosis resolved. In cases ketosis was associated with signs of dull demeanor affected animals were considered to be clinically ketotic and were administered 500 mL of 30% dextrose solution intravenously once or in 48h intervals in combination with oral propylene glycol at the dosage described above until symptoms resolved.

Blood and liver tissue collection. Blood samples were obtained by venipuncture of a jugular vein three times per week between 0700 and 0800. Ten mL of whole blood per sampling time were collected into a blood tube containing lithium-heparin as anticoagulant (Vacuette, Greiner Bio-One, Kremsmünster, Austria) and was centrifuged within 30 min of collection at 1000xg for 15 min at 6°C. Harvested plasma was stored at -21°C until analyzed as described below.

Liver tissue specimens were obtained at the time points T_1_, T_2_, T_3_, T_4_ and for cows of replicate 1 also at T_5_ using a micro-invasive biopsy technique. The procedure was conducted under local anesthesia (Procaine hydrochloride 4%, V.M.D. n.v. Arendonk, Belgium) and after administration of a non-steroidal anti-inflammatory drug (Metacam 20 mg/ml, Boehringer Ingelheim, Ingelheim am Rhein, Germany, 0.5 mg/kg s.c.). Liver tissue was obtained transcutaneously from the right 10^th^ intercostal space using an automated biopsy sampling device (BIP-Evocore EC2215, BIP Biomed. Instrumente und Produkte GmbH, Türckenfeld, Germany) armed with a 12G biopsy needle (BIP-Evocore EC12200, BIP Biomed. Instrumente und Produkte GmbH, Türckenfeld, Germany). The biopsy site was shaved over an area of 5 x 5 cm, anesthetized and surgically prepared before a small stab incision through the skin and the fascia of the intercostal muscle was placed. The biopsy needle was inserted through the incision and forwarded approximately 10 cm in cranio-ventral direction into the liver. By pulling the instrument trigger a stylet previously secured inside the biopsy needle was thrusted 2 cm further into the tissue, exposing a specimen notch. The needle sheath immediately followed the stylet thereby covering the specimen notch again and trapping liver tissue in it. The instrument was then gently retracted to harvest the obtained specimen. Two specimens, each of approximately 0.1g were obtained at each sampling time. Collected liver tissue was immediately placed in liquid nitrogen and then stored at -80°C until processed as described below.

#### Analyses

Plasma biochemical analysis. Plasma of all blood sampling times was analyzed for the concentration of [Pi] (ammonium molybdate method). Samples obtained at the time points T_1_ to T_5_ were furthermore analyzed for non-esterified fatty acids ([NEFA], ACS-ACOD method), betahydroxybutyric acid ([BHBA], UV-method), total bilirubin ([TBil], DCA method), and the enzyme activities of aspartate aminotransferase (AST, UV-method without pyridoxal phosphate), gammaglutamyl transferase (γGT, modified Szasz photometric method), and glutamate dehydrogenase (GLDH, α-ketoglutarate method). Plasma biochemical analyses were conducted on an automated analyzer (ABX Pentra 400; Horiba, Europe GmbH, Langenhagen, Germany).

Plasma samples obtained at T_1_ and T_2_ were assayed for the concentration of Insulin-like Growth Factor-I (IGF-1, IRMA, Beckman Coulter, Brea CA, USA).

Liver tissue biochemical analysis. Liver tissue DM of each specimen was determined by atmospheric oven drying to constant weight at 85°C as described earlier (Grünberg et al. 2014). The P-, potassium (K) and magnesium (Mg) content of each sample was determined in the previously dried specimens by inductively coupled optical emission spectrometry (ICP/OES, Varian Vista Pro, Darmstadt, Germany). The detected emission wave lengths were 185.878 nm for P, 766.491 nm for K and 279.553 nm for Mg. Potassium and Mg are like P predominantly intracellular electrolytes and were analysed to determine if changes in liver tissue P content over time were specific to P or also occurred with other intracellular electrolytes.

For lipid isolation, 5% homogenates of liver tissue were made in ice cold water using an ultra-turrax homogenizer. To 100μl of this homogenate, 900μl of chloroform / methanol (1/1; v/v) was added together with 5.0nmol sitosterol which was used as an internal standard. After mixing, samples were left to stand for 30 minutes at room temperature. After centrifugation for 5 min at 2000x*g* to remove the precipitate, 10μl aliquots of the supernatant were injected directly onto a Kinetex/HALO C8-e column (2.6 μm, 150 × 3.00 mm; Phenomenex, Torrance, CA, USA). Gradient elution was performed from methanol / water (1/1; v/v) to methanol / isopropanol (4/1; v/v) in 2 minutes, followed by isocratic elution with the latter solvent for an additional 7 minutes. Flow rate was kept constant at 600μl/min and a 1 minute re-equilibration time was used between runs. Mass spectrometry of eluting lipids was performed using positive mode Atmospheric Pressure Chemical Ionization (APCI) on a LTQ-XL mass spectrometer (Thermo, Waltham, MA, USA) in the range from 200–1100 Da [25].

The contents of P, K and Mg in dry liver tissue (P_DM_, K_DM_ and P_DM_ respectively) were used together with the liver tissue DM to derive the content of P, K and Mg in the liver on a wet weight basis (P_WW_, K_WW_ and Mg_WW_ respectively). Similarly, the electrolyte content in liver tissue was expressed on a TAG-free wet weight basis (P_TF_, K_TF_ and Mg_TF_ respectively) by subtracting the liver TAG from liver wet weight and expressing the content of each electrolyte as a fraction of this TAG-fee wet weight. Removal of the TAG weight that does not contribute to the volume of distribution of water soluble intracellular electrolytes was anticipated to produce a better estimate of the electrolyte concentrations in the hepatocytic cytosol [26].

Analyses of mRNA expression. To assess the activity of the carbohydrate metabolism the relative abundance of mRNA encoding cPEPCK, PC, PFKL were again studied as described for the in-vitro study described above. Acetyl CoA carboxylase (ACC) and sterol regulatory element binding factor 1 (SREBF1) were studied to assess effects of P deprivation on the lipid- and cholesterol metabolism, and mRNA of the gene encoding albumin (Alb) were determined as indicators for liver protein metabolism (S2 Table).

### Statistical analysis

Data presented in text and tables are expressed as least square means (LSM) ± SEM, LSM and 95% confidence interval for data log transformed for analysis, or median and interquartile range for data ranked for statistical analysis. Figures present data as mean and standard deviation (SD) or median and interquartile range. Values were log transformed when necessary to achieve normal distribution; if normal distribution could not be achieved by transformation results were ranked for analysis. For part 1 analyses of variance with an autoregressive(1) covariance matrix were used to identify treatment-, time- and treatment x time interaction effects. For part 2 repeated measures analyses of variance with the same covariance matrix with animal ID as repeated subject nested into treatment were used to determine effects of replicate-, treatment-, time-, the interactions between treatment x time and between replicate x treatment using PROC MIXED (SAS 9.4, SAS Inc, Cary NC). The autoregressive(1) covariance structure was chosen based on the lowest Akaike information criterion. The significance level was set at *P*<0.05. Bonferroni-adjusted *P* values were used to assess differences between time points whenever the *F* test was significant.

Pearson or Spearman correlation analyses and multiple stepwise regression analyses were conducted to identify possible associations between liver biochemical parameters and relative abundance of studied enzymes in liver, between liver biochemical parameters and blood biochemical parameters from samples obtained at the time of collection of liver tissue specimens as well as between relative mRNA abundances of liver enzymes and blood biochemical parameters obtained at the corresponding time. Correlation and regression analyses were conducted for each sampling time separately. Parameters were included as independent variables in the stepwise regression analysis with a *P* = 0.2 as entry and *P* = 0.05 as exit. The final regression models were checked for variance inflation by screening tolerance, variance inflation factors, and the eigenvalue in combination with the condition index of each variable in the final model. In case of suspected multicolinearity between some variables in the final model, the variable potentially affected by colinerarity with the lowest R^2^ was removed and the analysis rerun. All analyses were conducted with SAS software (SAS 9.4, SAS Inst. Inc. Cazy, NC).

## Results

### In-vitro study

**Fig 2:**
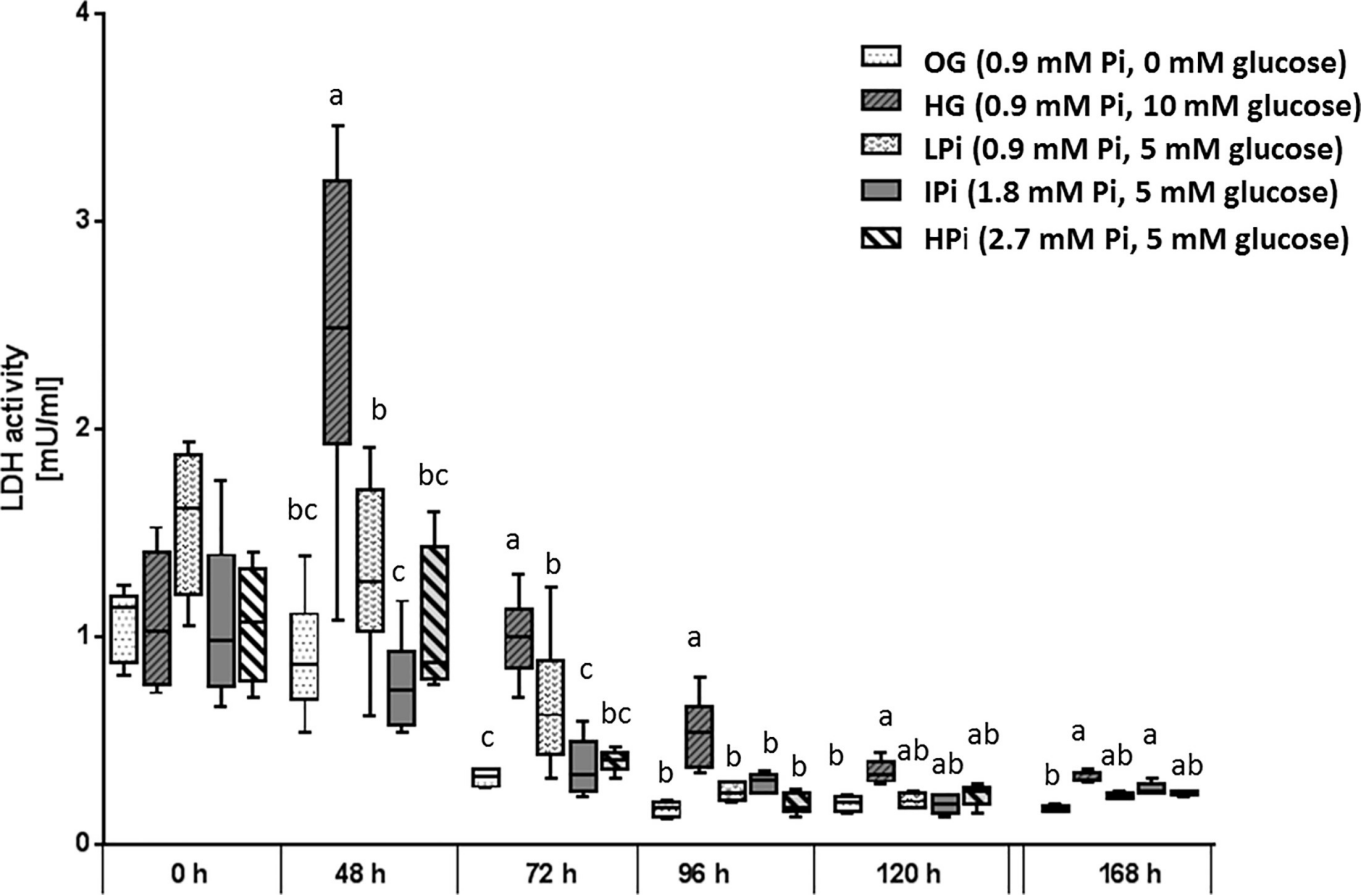
LDH activity in culture medium of primary bovine hepatocytes cultured with different phosphate (Pi) concentrations and glucose levels for seven days in sandwich culture between two layers of collagen. Culture medium was replaced initially 6h after onset of incubation (0h) then 24h after onset of incubation and in 24h intervals thereafter. Medium obtained at 48h and later after onset of incubation was thus exposed to cultured cells for the same time interval (24h). Box and whisker plots represent median (horizontal line), lower and upper quartiles (bottom and top of box respectively) as well as 2^nd^ and 9^th^ percentile (lower and upper end of whiskers respectively). Treatments with different lower case letters at one sampling time differ significantly from each other (*P*<0.05, Bonferroni corrected).

Staining with trypan blue of harvested cells indicated cell viability was 77.0% at the onset of incubation. The LDH activity in culture medium showed significant treatment-, time- and treatment x time interaction effects (all *P*<0.0001*)*.The LDH activity declined in all experimental media between 24h and 72h of incubation and remained constant thereafter until the end of the study at 168h (Fig 2). LDH activities in HG medium were higher than in 0G medium at all sampling times. LDH activities of LPi, IPi and HPi media ranged between activities determined in HG and 0G. Differences between LPi, IPi and HPi were only significant when stratified by time (treatment x time interaction) with higher LDH activities in LPi compared to IPi during the early phase of incubation, at the sampling times 48h and 72h (Fig 2).

**Fig 3:**
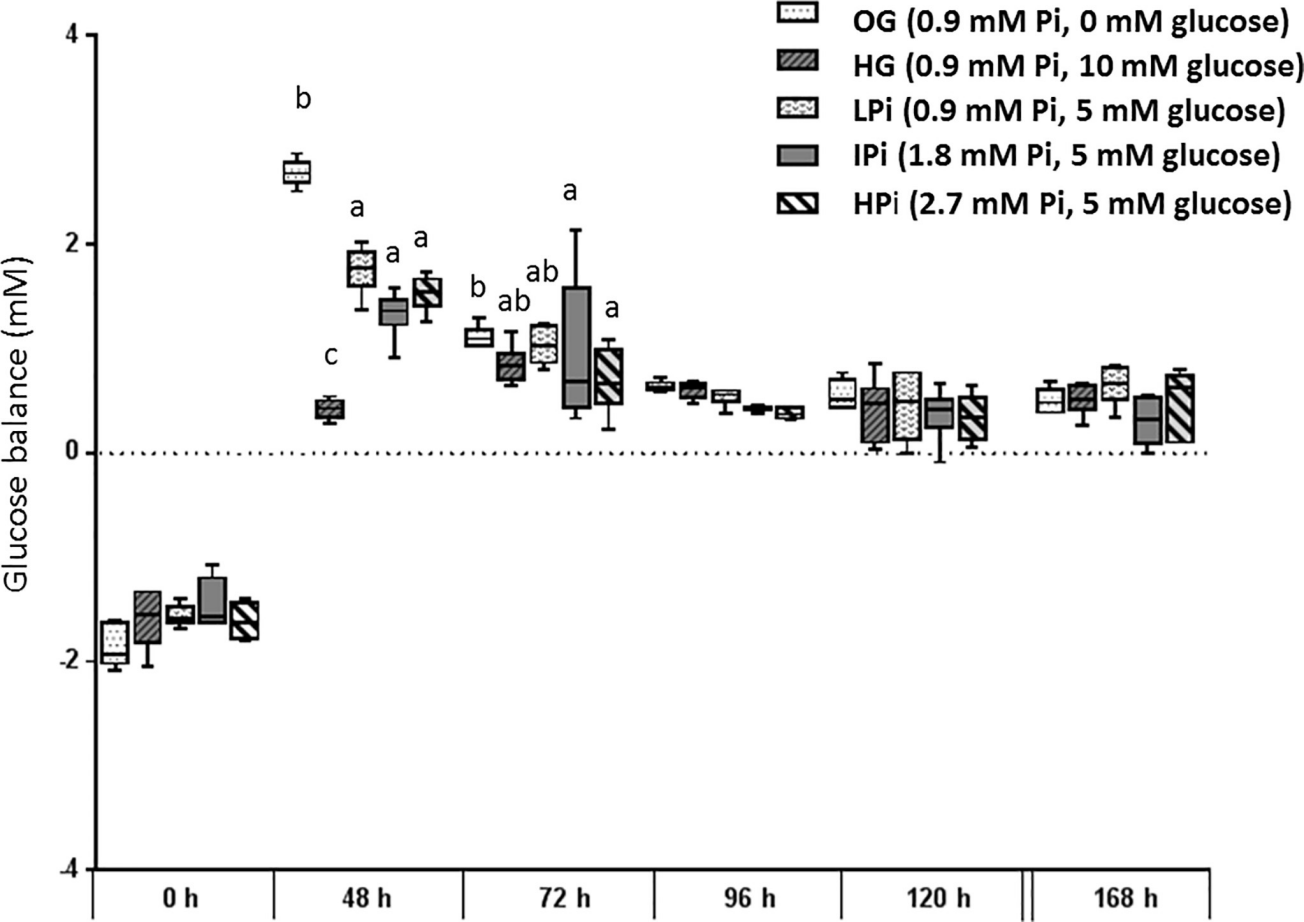
Glucose balance of primary bovine hepatocytes cultured with different phosphate (Pi) concentrations and glucose levels for seven days in sandwich culture between two layers of collagen. Culture medium was replaced initially 6h after onset of incubation (0h) then 24h after onset of incubation and in 24h intervals thereafter. Medium obtained at 48h and later after onset of incubation was thus exposed to cultured cells for the same time interval (24h). Box and whisker plots represent median (horizontal line), lower and upper quartiles (bottom and top of box respectively) as well as 2^nd^ and 9^th^ percentile (lower and upper end of whiskers respectively). Groups with different lower case letters at one sampling time differ significantly from each other (*P*<0.05, Bonferroni corrected).

Significant treatment-, time- and treatment x time interaction effects (all *P*<0.0001) were identified for the glucose balance. The treatment effect consisted in significant differences between all treatments with exception of IPi that did neither differ from LPi nor HPi. LPi medium however had a more positive glucose balance than HPi medium (0.40 ± 0.03 mmol/L vs. 0.54 ± 0.03 mmol/L; *P* = 0.003). Most pronounced differences were found between 0G and HG with 0G-medium having the highest (0.84 ± 0.02 mmol/L) and HG medium the lowest (0.08 ± 0.03 mmol/L) glucose balance, an effect that was particularly apparent from 6h to 48h of incubation (Fig 3).

**Fig 4:**
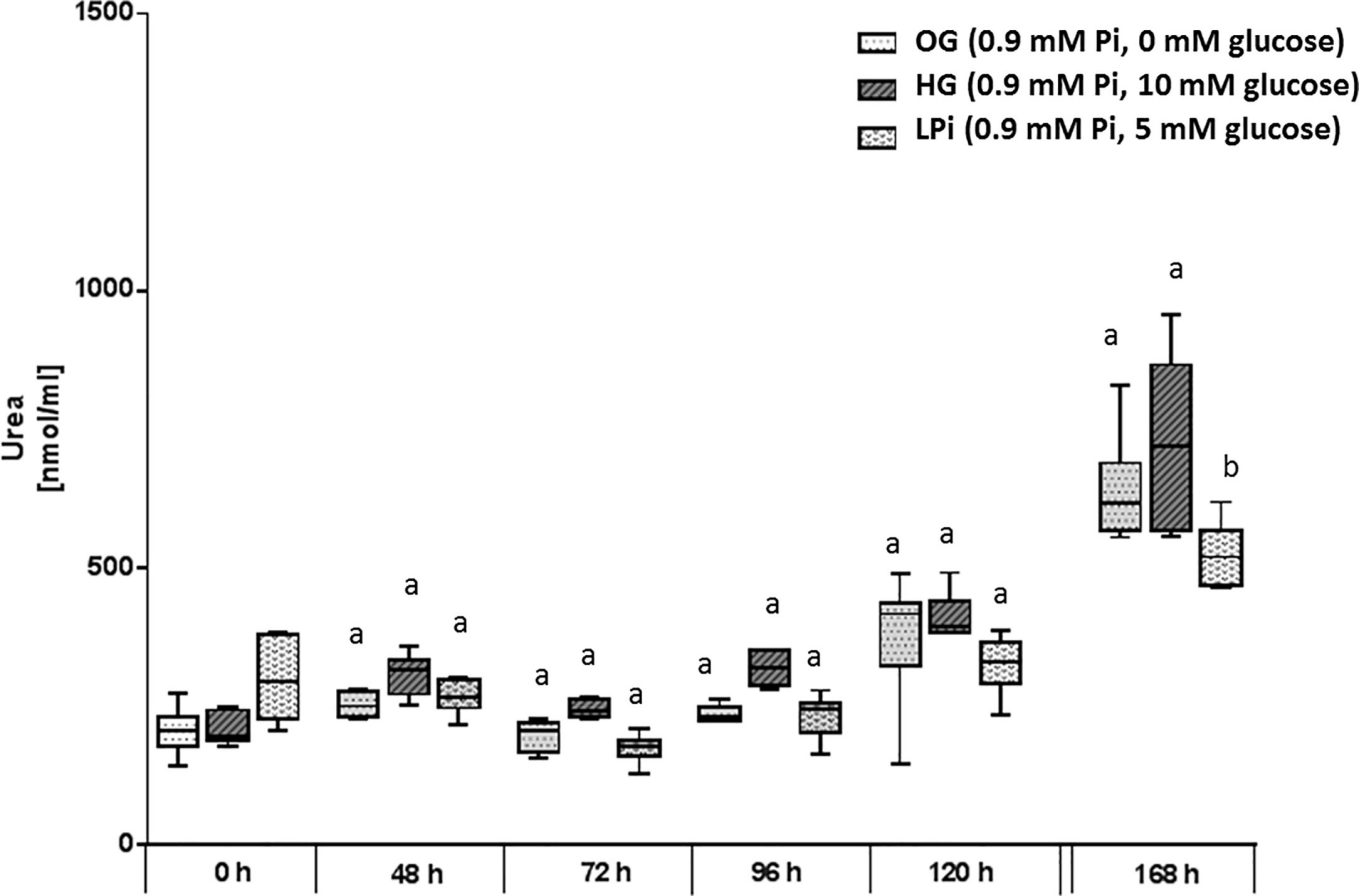
Urea concentration in culture medium of primary bovine hepatocytes cultured at different glucose concentrations with 0.9 mmol/L [Pi] for seven days in sandwich culture between two layers of collagen. Culture medium was replaced initially 6h after onset of incubation (0h) then 24h after onset of incubation and in 24h intervals thereafter. Medium obtained at 48h and later after onset of incubation was thus exposed to cultured cells for the same time interval (24h). Box and whisker plots represent median (horizontal line), lower and upper quartiles (bottom and top of box respectively) as well as 2^nd^ and 9^th^ percentile (lower and upper end of whiskers respectively). Groups with different lower case letters at one sampling time differ significantly from each other (*P*<0.05, Bonferroni corrected).

Urea concentrations in medium that was only determined in 0G-, HG- and LPi media also revealed a significant treatment- (*P* = 0.0021), time- (*P*<0.0001) and treatment x time interaction effect (*P* = 0.0005; Fig 4). Urea concentrations were higher in HG- compared to 0G and LPi medium. The urea concentration increased markedly over time but to a similar degree in all three media (Fig 4).

#### Relative abundance of specific mRNA in liver tissue

The results of the relative abundance analysis of mRNA encoding specific liver enzymes and the transcription of albumin are presented in Table 2. Significant time effects were identified for all of the studied parameters with exception of cPEPCK. A marked increase was observed for the relative abundance of all enzymes between 6h and 72h with the exception of PFKL where values were lower after 72h than after 6h of incubation (Table 2). Treatment effects were observed for cPEPCK with lower values for HG than for all other treatments, for PFKL with higher values for LPi than 0G, and for NFkBIa with lower values for 0G than for LPi, IPi and HPi (Table 2).

**Table 2:** Results of the analysis of variance of the relative abundances of specific mRNA in primary bovine hepatocytes incubated with different experimental media.

Item	Time		Treatment (Medium)					Effects		
	h		0G	HG	LPi	IPi	HPi	Tx	Time	Tx • Time
**cPEPCK**	**all**		0.05^a^ [0–2.16]	0.03^b^ [0–2.11]	0.04^a^ [0–2.10]	0.05^a^ [0–2.16]	0.05^a^ [0–2.26]	<0.0001	N.S	N.S
	**6**	0.04 [0–2.10]	0.04^a^ [0–2.23]	0.03^b^ [0–2.16]	0.04^a^ [0–2.15]	0.04^a^ [0–2.15]	0.06^a^ [0–2.38]			
	**72**	0.04 [0–2.10]	0.05^a^ [0–2.23]	0.03^b^ [0–2.15]	0.04^ab^ [0–2.14]	0.06^b^ [0–2.23]	0.04^ab^ [0–2.36]			
**PC**	**all**		0.27 [0–2.42]	0.24[0–2.38]	0.30[0–2.40]	0.28 [0–2.41]	0.30[0–2.40]	N.S	<0.0001	N.S
	**6h**	0.14* [0–2.23]	0.15* [0–2.39]	0.12* [0–2.36]	0.17* [0–2.33]	0.14* [0–2.34]	0.15* [0–2.51]			
	**72**	0.51[0–2.62]	0.63[0–2.72]	0.47[0–2.68]	0.54[0–2.68]	0.57[0–2.78]	0.63[0–2.99]			
**PKL**	**all**		0.15 + 0.01	0.16 + 0.01	0.15 + 0.01	0.16 + 0.01	0.15 + 0.01	N.S	<0.0001	N.S
	**6**	0.09* + 0.01	0.09*+ 0.01	0.11* + 0.02	0.10* + 0.01	0.09* + 0.01	0.08* + 0.01			
	**72**	0.22 + 0.01	0.22 + 0.01	0.21 + 0.02	0.20 + 0.01	0.23 + 0.01	0.21 + 0.01			
**PFKL**	**all**		0.90^a^ [0–3.84]	0.92^ab^ [0–2.99]	1.09^b^ [0–3.16]	0.95^ab^ [0–3.04]	1.05^ab^ [0–3.12]	0.05	<0.0001	N.S
	**6**	1.17* [0–3.20]	1.04 [0–3.12]	1.12 [0–3.24]	1.41 [0–3.53]	1.11 [0–3.25]	1.22 [0–3.34]			
	**72**	0.82 [0–2.84]	0.78 [0–2.85]	0.75 [0–2.87]	0.17 [0–2.29]	0.81 [0–2.69]	0.90 [0–3.02]			
**Alb**	**all**		0.09 + 0.01	0.09 + 0.01	0.12 + 0.01	0.10 + 0.01	0.10 + 0.01	N.S	<0.0001	N.S
	**6**	0.07* + 0.00	0.07* + 0.01	0.06* + 0.01	0.07* + 0.01	0.06* + 0.01	0.07* + 0.01			
	**72**	0.14 + 0.01	0.11 + 0.01	0.13 + 0.01	0.16 + 0.01	0.15 + 0.01	0.13+ 0.01			
**HNF4a**	**all**		1.16 [0–3.27]	1.09 [0–3.16]	1.12 [0–3.19]	1.20 [0–3.27]	1.23 [0–3.40]	N.S	<0.0001	N.S
	**6**	0.98* [0–3.03]	1.00 [0–3.17]	0.93* [0–3.05]	1.01 [0–3.14]	1.00* [0–3.17]	0.96* [0–3.23]			
	**72**	1.37 [0–3.42]	1.35 [0–3.52]	1.28 [0–3.38]	1.24 [0–3.36]	1.45 [0–3.57]	1.56 [0–3.83]			
**NFkBIa**	**all**		1.18^a^ ± 0.05	1.25 ± 0.06^ab^	1.44 ± 0.06^b^	1.42 ± 0.07^b^	1.42^b^ ± 0.05	0.003	<0.0001	N.S
	**6**	0.78* ± 0.038	0.79 ± 0.07	0.87 ± 0.08	1.00 ± 0.09	0.86 ± 0.11	0.88 ± 0.07			
	**72**	1.80 + 0.038	1.58^a^ ± 0.07	1.63^ab^ ± 0.08	1.88^b^ ± 0.09	1.97^b^ ± 0.11	1.96^b^ ± 0.07			

Least square means and SEM or 95% confidence intervals of relative abundance of liver enzymes stratified by time (6h and 72h) and by treatment (0G: no glucose, [Pi] = 0.9 mmol/L; HG: [glucose] = 10 mmol/L, [Pi] = 0.9 mmol/L; LPi: [glucose] = 5mmol/L; [Pi] = 0.9 mmol/L; IPi: [glucose] = 5mmol/L; [Pi] = 1.8 mmol/L; HPi: LPi: [glucose] = 5 mmol/L; [Pi] = 2.7 mmol/L). Values with different superscript letters differ significantly between treatment (*P*<0.005, Bonferroni corrected); values with asterisk (*) within one column differ significantly between times.

### Clinical study

#### General health

Cows assigned to LP and C were 4.7 ± 0.6 and 4.5 ± 0.6 years old respectively and in their 3^rd^ ± 1 lactation. All 36 cows delivered healthy calves either spontaneously or with mild to moderate assistance. Four cows, all assigned to treatment LP were excluded from the study 2 to 4 weeks after parturition, thus between one and two weeks after T_3_. Three of these cows developed postparturient hemoglobinuria during the second week of lactation, that was characterized by severe intravascular hemolysis and ensuing profound anemia and hemoglobinuria. One dam of LP with twins retained her fetal membranes and later developed metritis and clinical ketosis and was excluded due to ongoing deterioration. Results from these cows obtained up to the first week of lactation (T_3_) were retained in the dataset as no clinical or clinicopathological abnormalities were apparent until then. Two cows, one of each treatment were diagnosed with a left displaced abomasum in the 2^nd^ week of lactation. Both cows underwent surgical correction of the abomasal displacement and made an uneventful recovery and completed the study. Clinical ketosis was diagnosed in 1 C and 2 LP cows and was treated as described above. Intravenous dextrose treatment was never administered in the 48h before liver tissue sample collection; blood samples collected within 48h following dextrose infusion were excluded from analysis. Periparturient hypocalcemia was diagnosed clinically and confirmed by blood biochemical analysis in 4 dams of treatment C but none of treatment LP. All of these cows made an uneventful recovery after parenteral and oral administration of calcium salts. The calcium homeostasis of a subset of these animals was studied and reported elsewhere [20].

#### Feed intake and milk production

**Fig 5:**
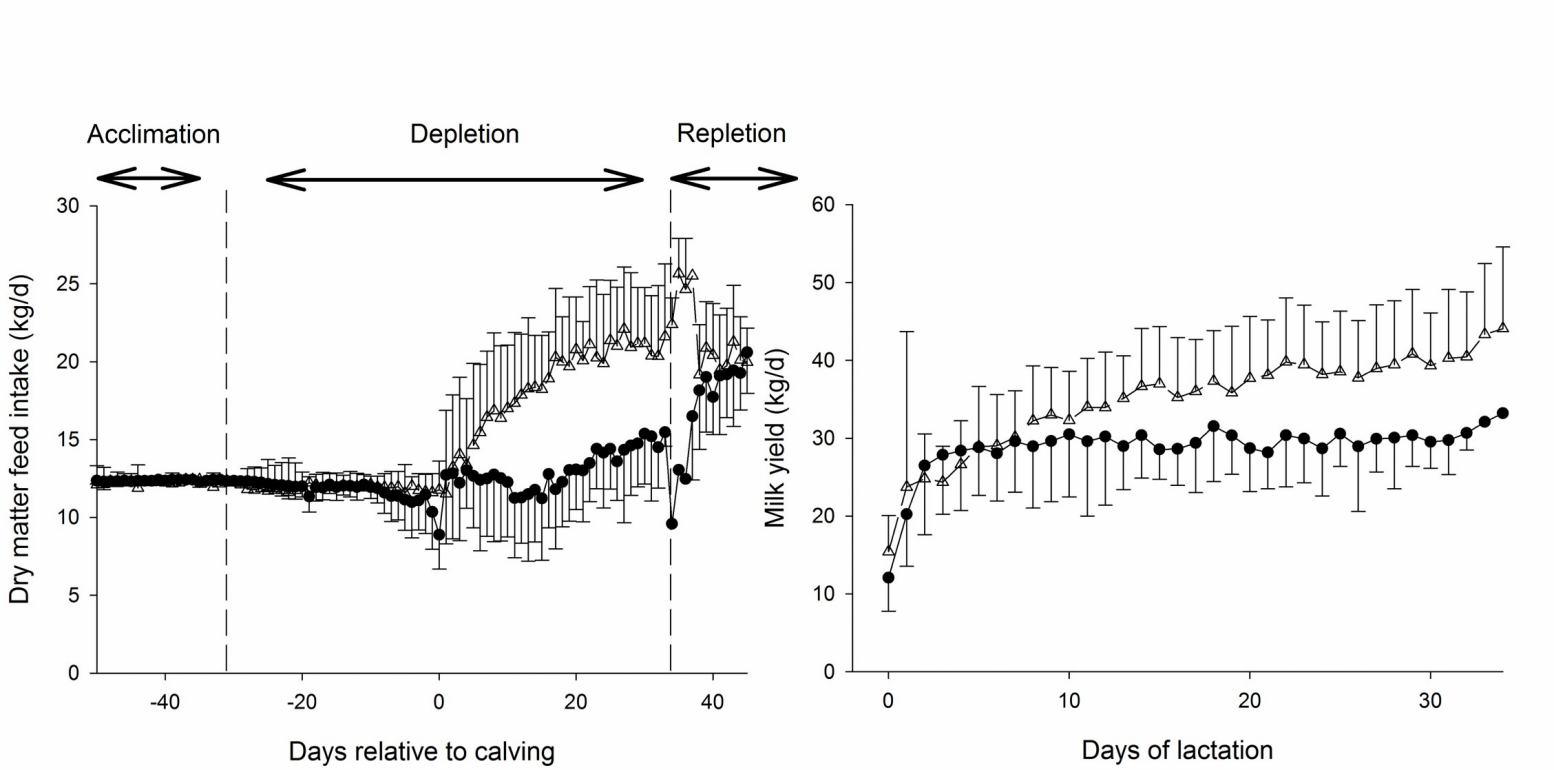
Feed intake and milk yield. Mean daily dry matter feed intake (left panel) and milk production (right panel) of cows undergoing dietary P depletion (LP, closed circles, solid line) and control cows on a diet with adequate P content (C, open triangles, dashed line). The vertical dashed lines in the right panel denote begin and end of the dietary P-depletion period.

Daily feed intake and milk production for both treatments are presented in Fig 5. Feed intake did not differ between groups during the dry period (restricted feeding) and the first week of lactation. Differences in feed intake between groups became apparent from the second week of lactation with an approximately 20% lower feed intake from the second to the fourth week of lactation in LP compared to C (Fig 5). The typical increase in milk production of early lactating of LP cows lagged approximately 20% behind the production of C animals (Fig 5).

#### Blood biochemical analysis

**Fig 6:**
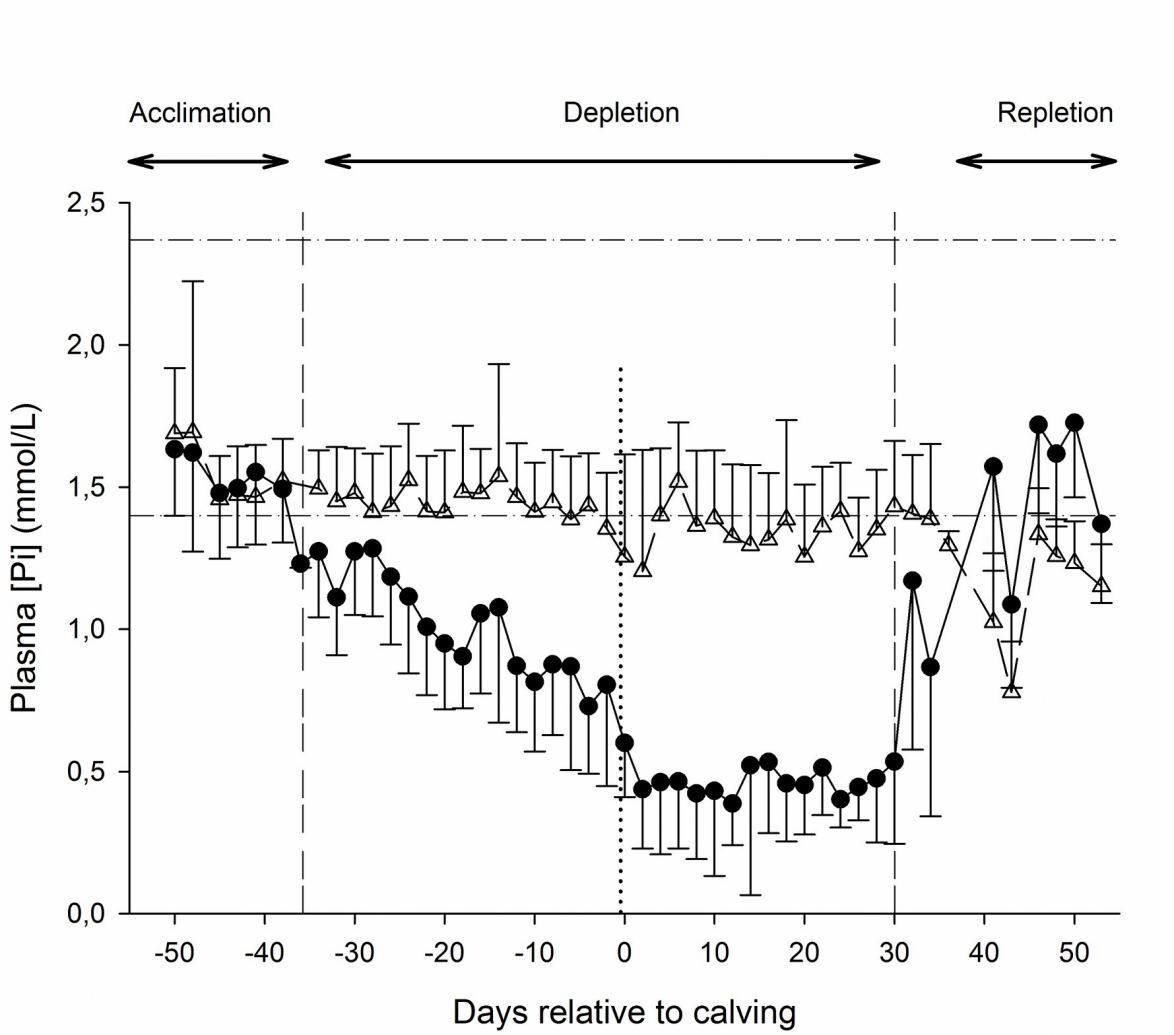
Plasma phopshate concentrations. Mean ± SD of the plasma phosphate concentration ([Pi]) of cows undergoing dietary P deprivation (LP, closed circles, solid line) and control cows on a diet with adequate P content (C, open triangles, dashed line). The vertical dashed lines in the right panel denote begin and end of the dietary P-depletion period. Horizontal dashed lines mark upper and lower reference range for plasma phosphate in adult cattle. Values of all sampling times of the the P-depletion period differed significantly between treatments (*P*<0.05). Values of all sampling times of the depletion period were signifcantly lower than sampling times of the acclimation and repletion period (*P* < .0.05, Bonferroni corrected).

The concentration—time curves for plasma [Pi] stratified by treatment are presented in Fig 6. Significant treatment-, time- and treatment x time interaction effects (all *P*<0.0001) were identified, with plasma [Pi] for LP below values of C during the entire deprivation period. The mean plasma [Pi] of LP cows was below values measured during the acclimation period and below the reference range for [Pi] in cattle (1.4–2.3 mmol/L) during the entire deprivation period (Fig 6) [27]. Values did not differ between treatments during the repletion period of the study (Fig 6).

The results of the analysis of liver specific plasma biochemical parameters determined at the sampling times T_1_ to T_5_ are summarized in Tables 3 and 4. Time effects were significant for all investigated parameters and characterized by pronounced increases after parturition. The only parameter for which a weak but significant treatment effect was identified was γGT with slightly lower values in LP cows than in C animals (Table 4). This result was however conflicting in both replicates (replicate x treatment interaction effect) with higher values in C compared to LP in the first replicate but the opposite relation in the second replicate (Table 4). A treatment x time interaction effect was only identified for TBil with higher concentrations in LP than in C cows at T_3_ (Table 4). Replicate effects were observed for AST and BHBA with higher values in replicate 2 than in replicate 1. Neither the correlation- nor the stepwise regression analyses revealed associations of plasma [Pi] with liver specific plasma biochemical parameters (Table 3).

**Table 3:** Results of the repeated measures analysis of variance (*P* values) for liver specific plasma biochemical parameters.

Item	Effects				
	Tx	Time	Replicate	Tx • Time	Tx • Replicate
**AST**	N.S	<0.0001	0.0002	N.S	N.S
**GLDH**	N.S	0.0021	N.S	N.S	N.S
**γGT**	0.0473	0.0344	N.S	N.S	0.0122
**TBil**	N.S	<0.0001	N.S	0.0431	N.S
**NEFA**	N.S	<0.0001	N.S	N.S	N.S
**BHBA**	N.S	<0.0001	0.0260	N.S	N.S

Studied parameters were the activities of aspartate aminotransferase (AST), glutamate dehydrogenase (GLDH), gamma glutamyl transferase (γGT), and the plasma concentrations of total bilirubin (TBil), non-esterified fatty acids (NEFA) and betahydroxybutyric acid (BHBA). Studied effects were treatment (Tx), time, replicate, Tx • Time and Tx • Replicate interactions. N.S: No significant effect.

**Table 4:** Results of the analysis of variance of liver specific plasma biochemical parameters.

Item	Tx	Sampling time					Treatment	Replicate	
		T_1_	T_2_	T_3_	T_4_	T_5_		1	2
**AST**	LP	55.0^a^ [52.9–57.0]	55.0^a^ [52.9–57.0]	75.9^b^* [73.8–77.9]	72.4^b^ [70.3–74.5]	81.3^b^ [79.-83.4]	67.6 [65.6–69.6]	64.6^a^ [62.5–66.6]	72.4^b^ [72-1-76.2]
(IU/L)	C	55.0^a^ [52.9–54.7}	58.9^a^ [56.8–60.9.0]	87.1^b^ 85.0–89.2]	74.1 ^b^ [72.1–76.2]	75.9^b^ [73.8–76.2]	69.2 [67.2–71.2]	61.7^a^ [59.6–63.7]	74.1^b^ [72.1–76.2]
**GLDH**	LP	10.0^ab^ [7.8–12.2]	10.0^ab^ [7.8–12.2]	7.9^a^ [5.7–10.1]	12.6^b^ [10.4–14.8]	10.0^ab^ [7.8–12.2]	10.0 [7.6–12.4]	12.6 [10.5–14.7]	10.0 [7.9–12.2]
(IU/L)	C	12.6^ab^ [10.4–14.8]	10.0^a^ [7.8–12.2]	10.0^a^ [7.8–12.2]	15.8^b^ [13.7–18.1]	10.0^ab^ [7.7–12.3]	11.2 [9.2–13.3]	12.6 [10.5–14.7]	10.0 [7.9–12.2]
**γGT**	LP	20.0 [17.8–22.1]	19.0 [16.9–21.2]	18.2 [16.1–20.3]	19.5 [13.3–21.6}	23.4 [21.2–25.6]	20.0* [17.9–22.0]	19.5^a^* [17.4–21.6]	27.5^b^ [25.3–29.7]
(IU/L)	C	21.9 [19.8–24.0]	20.0 [17.8–22.1]	19.5 [17.4–21.6]	24.0 [21.9–26.1]	27.5 [25.3–29.7]	22.4 [20.3–24.4]	25.1^a^ [23.1–27.2]	20.4^b^ [18.3–22.5]
**TBil**	LP	3.3^a^ [1.2–5.5]	3.7^a^ [1.6–5.8]	9.1^b^* [7.0–11.0]	5.3^c^ [3.1–7.5]	3.7^ac^ [1.5–6.0]	4.7 [2.6–6.7]	4.7 [2.6–6.8]	3.7 [1.5–6.0]
(μmol/L)	C	3.5^a^ [1.4–5.7]	3.6^a^ [1.4–5.7]	6.0^b^ [3.8–8.1]	4.4^a^ [2.3–6.5]	4.1^a^ [1.9–6.3]	4.2 [2.15–6.24]	4.3 [2.3–6.4]	4.6 [2.5–6.7]
**NEFA**	LP	100^a^ [98–102]	115^a^ [113–117]	562^b^ [560–565]	309^c^ [307–311]	240^c^ [238–242]	219 [217–221]	224 [222–226]	240 [238–242]
(mmol/L)	C	93^a^ [91–96]	100^a^ [98–102]	537^b^ [535–539]	275^c^ [273–278]	398^bc^ [396–400]	224 [222–226]	214 [212–216]	2.33 [212–216]
**BHBA**	LP	0.47^a^ [0–2.66]	0.44^a^ [0–2.62]	0.58^ab^ [0–2.77]	0.75^b^ [0–2.97]	0.63^ab^ [0–2.98]	0.56 [0–2.64]	0.50 [0–2.61]	0.64 [0–2.78]
(mmol/L)	C	0.44^a^ [0–2.63]	0.44^a^ [0–2.63]	0.74^b^ [0–2.93]	0.78^b^ [0–2.96]	0.58^ab^ [0–2.89]	0.58 [0–2.65]	0.55 [0–2.65]	0.61 [0–2.74]

Activities of aspartate aminotransferase (AST), glutamate dehydrogenase (GLDH), gamma glutamyl transferase (γ-GT), and the plasma concentrations of total bilirubin (TBil), non-esterified fatty acids (NEFAs) and betahydroxybutyrate (BHBA) are presented as least square means and 95% confidence interval, stratified by sampling times T_1_ (end of acclimation), T_2_ (after two weeks of dietary P deprivation), T_3_ within the first week of lactation, T_4_ (end of deprivation) and T_5_ (only for cows of replicate 1, after two weeks of repletion), by treatment (LP: low phosphate and C: control) and by replicate number. Values with different superscript letters within a row differ significantly (*P*<0.05, Bonferroni corrected). Values with asterisk (*) differ between treatments for the specific time point.

Plasma IGF-1 concentrations measured at T_1_ and T_2_ were 179.0 ± 13.2 and 228.1 ± 12.8 ng/mL respectively for treatment C and 175.9 ± 12.8 and 211.6 ± 12.8 ng/mL respectively for treatment LP. No treatment-, time or treatment x time interaction effects was observed. A significant replicate effect (*P* = 0.0016) with lower values in replicate 1 (177.5 ± 9.2 ng/mL) than replicate 2 (219.8 ± 9.0 ng/mL) was identified. The correlation analyses identified a significant association between plasma [Pi] and plasma IGF-I at T_1_ (r = 0.41; *P* = 0.015) and at T_2_ (r = 0.40; *P* = 0.017). The stepwise regression analysis with plasma IGF-1 as dependent variable and treatment, replicate, and plasma [Pi] as independent variable stratified by sampling time only revealed an association with plasma [Pi] (partial R^2^ = 0,125; *P* = 0.03) at T_1_.

#### Liver biochemical analysis

Results of the liver tissue dry matter analysis are presented in Table 5. The analysis of variance revealed a significant time effect with DM increasing between T_2_ and T_4_ as well a significant replicate effect with liver tissue DM of replicate 1 approximately 4% above values of replicate 2 (Table 5). Neither a significant treatment nor a treatment x time interaction effect was identified.

**Table 5:** Results of the analysis of variance of liver tissue biochemical parameters.

Item	Tx	Sampling time						Treatment		Replicate			Effects (*P* value)			
		T_1_	T_2_	T_3_	T_4_	T_5_	SEM	LSM	SEM	1	2	SEM	Tx	Time	Replicate	Tx • time
**P**_**ww**_	LP	2.97^a^	2.74^ab^*	3.04^a^	2.56^b^	2.44^b^*	0.10	2.75	0.04	2.98	2.51	0.05	0.0023	<0.0001	<0.0001	0.0182
^(g/kg)^	C	2.92^ab^	3.13^a^	3.00^ab^	2.77^b^	2.85^ab^	0.08	2.93	0.04	3.07	2.79	0.06				
**P**_**Tf**_	LP	2.95^ab^	2.75^a^*	3.17^b^	2.85^a^	2.76^ab^	0.08	2.90	0.04	3.12^a^	2.67^b^*	0.06	0.0101	0.0097	<0.0001	0.0304
^(g/kg)^	C	2.92	3.19	3.15	2.95	3.08	0.08	3.06	0.04	3.20^a^	2.91^b^	0.06				
**P**_**DM**_	LP	9.9	9.7	10.0	8.0	8.2	0.3	9.2	0.2	9.2*	9.2*	0.2	0.0002	<0.0001	N.S.	N.S
^(g/kg)^	C	10.0	10.9	10.3	9.2	9.7	0.3	10.0	0.2	10.0	10.0	0.2				
**K**_**ww**_	LP	2.23^ab^	2.09^a^*	2.30^b^	2.06^a^	1.99^a^	0.06	2.13	0.03	2.32^a^	1.95^b^	0.04	N.S	0.0028	<0.0001	N.S
^(g/kg)^	C	2.27^a^	2.27^a^	2.21^ab^	2.06^b^	2.17^ab^	0.05	2.19	0.03	2.33^a^	2.05^b^	0.04				
**K**_**Tf**_	LP	2.25^ab^	2.11^a^*	2.42^b^	2.30^ab^	2.16^ab^	0.06	2.25	0.03	2.44^a^	2.05^b^*	0.06	N.S	0.0291	<0.0001	0.0377
^(g/kg)^	C	2.30	2.31	2.32	2.20	2.25	0.05	2.27	0.03	2.43^a^	2.12^b^	0.06				
**K**_**DM**_	LP	7.5	7.4	7.6	6.4	6.7	0.3	7.1	0.1	9.2*	9.2*	0.2	0.0002	<0.0001	N.S.	N.S
^(g/kg)^	C	7.8	7.9	7.6	6.8	7.4	0.2	7.5	0.1	7.2	7.1	0.2				
**Mg**_**ww**_	LP	0.17^ab^	0.15^a^*	0.18^b^	0.15^a^	0.15^a^	0.00	0.16	0.00	0.17^a^	0.15^b^	0.00	0.0091	<0.0001	<0.0001	N.S
^(g/kg)^	C	0.17^ab^	0.17^a^	0.18^a^	0.16^b^	0.16^ab^	0.00	0.17	0.00	0.18^a^	0.16^b^	0.00				
**Mg**_**Tf**_	LP	0.16^a^	0.15^a^*	0.19^b^	0.16^a^	0.17^ab^	0.00	0.17	0.00	0.18^a^	0.15^b^	0.00	N.S	<0.0001	<0.0001	0.0479
^(g/kg)^	C	0.17^ab^	0.18^ab^	0.18^a^	0.17^b^	0.17^ab^	0.00	0.17	0.00	0.19^a^	0.16^b^	0.00				
**Mg**_**DM**_	LP	564	548	600	461	503	17	535	8	537*	533*	11	0.0018	<0.0001	N.S.	N.S
^(mg/kg)^	C	590	604	601	522	557	12	574	9	576	574	11				
**DM**	LP	29.4^ab^	28.1^a^	30.6^ab^	32.6^b^	30.4^ab^	0.8	30.2	0.4	32.5^a^	27.9^b^	0.6	NS	0.0048	<0.0001	N.S
^(%)^	C	29.2	28.7	29.5	30.8	29.8	0.8	29.6	0.4	31.2^a^	28.0^b^	0.5				

Liver tissue phosphorus, potassium and magnesium content in liver tissue wet weight (P_WW_, K_WW_ and Mg_WW_ respectively), in TAG-free wet weight (P_Tf_, K_Tf_ and Mg_Tf_ respectively) and dry matter (P_DM_, K_DM_ and Mg_DM_ respectively) as well as liver dry matter (DM, % of wet weight) are presented as least square means (LSM) and standard error (SEM). Studied effects were treatment (Tx), time, replicate and the interaction treatment x time. Sampling times are T_1_ (end of acclimation), T_2_ (after two weeks of dietary P deprivation), T_3_ within the first week of lactation, T_4_ (end of deprivation) and T_5_ (only for cows of replicate 1, after two weeks of repletion). Treatments are low phosphate (LP) and control (C). Values with different superscript letters within one line differ significantly (*P*<0.05, Bonferroni corrected); values with asterisk (*) differ between treatments within one row; Tx = treatment.

The liver P content showed a significant treatment- and time effect when expressed as P_WW_, P_Tf_ and P_DM_ with values approximately 22%, 5% and 8% lower in LP than in C for P_WW_, P_Tf_ and P_DM_ respectively. Treatment effects were also observed for K_DM,_ Mg_DM_ and Mg_WW_, with K_DM_ and Mg_DM_ of LP cows in the range of 5% below values of C cows. Time effects were found for all electrolyte-related parameters of the liver in form of significant declines between T_3_ and T_4_ (Table 5). Replicate effects were identified for P, K and Mg when expressed on a wet weight and TAG-free wet weight basis but not when expressed on DM-basis (Table 5).

The liver TAG contents stratified by sampling time and treatment are presented in Table 6. Treatment- (*P* = 0.0012), time- (*P*< 0.0001), treatment x time interaction (*P* = 0.0157) and a replicate effect (*P* = 0.0057) were observed. The liver TAG content rose between T_2_ and T_4_ for both treatments. In replicate 2, where values for T_5_ were also available a decline between T_4_ and T_5_ was observed. The liver TAG content was higher in LP than in C at T_4_ and T_5_ and higher in replicate 1 than replicate 2 (Table 6).

**Table 6:** Results of the analysis of variance of the liver triacylglycerol (TAG) content.

Item	Tx	Sampling time					Treatment	Replicate		Effects (*P* value)			
		T_1_	T_2_	T_3_	T_4_	T_5_		Replicate 1	Replicate 2	Tx	Time	Replicate	Tx • time
**TAG** ^mg/g^	LP	4.5^a^ [1.9–7.0]	5.2^a^ [2.7–7.6]	36.7^b^ [34.3–39.1]	80.3^c^* [77.9–82.8]	36.6^b^* [33.7–39.4]	19.0* [16.9–21.2]	21.1^a^* [18.9–23.4]	17.2^b^* [14.7–19.7]	0.0009	<0.0001	0.0084	0.0106
	C	4.9^a^ [2.4–7.4]	4.6^a^ [2.2–7.0]	35.8^b^ [33.4–38.1]	29.8^b^ [27.4–32.1]	7.7^a^ [5.0–10.5]	11.3 [9.1–13.5]	15.3^a^ [13.0–17.5]	8.4^b^ [6.1–10.7]				

Liver triacylglycerol (TAG) content is presented as LSM and 95% confidence interval. Studied effects were treatment (Tx), time, replicate and the interaction treatment • time. Sampling times are T_1_ (end of acclimation), T_2_ (after two weeks of dietary P deprivation), T_3_ within the first week of lactation, T_4_ (end of deprivation) and T_5_ (only for cows of replicate 1, after two weeks of repletion). Treatments are low phosphate (LP) and control (C). Values with different superscript letters within one line differ significantly (*P*<0.05, Bonferroni corrected); values with asterisk (*) differ between treatments within one row; Tx = treatment.

The results of the correlation analysis of electrolyte related parameters of the liver with the liver DM, liver TAG and DMI at the different sampling times are summarized in Table 7. Liver DM and liver TAG were strongly and positively correlated at T_3_, T_4_ and T_5_ but not at T_1_ and T_2_. The liver DM was furthermore negatively associated with DMI at T_1_, T_4_ and T_5_. For liver TAG a negative association with DMI was only observed at T_4_ that was considerably weaker than the association between liver TAG and DM at the same time point (Table 7).

**Table 7:** Results of the correlation analysis of liver tissue electrolyte contents with liver tissue triacylglycerol (TAG), liver dry matter content and (DM) dry matter intake (DMI).

	DM	DMI	P_ww_	P_Tf_	P_DM_	K_ww_	K_Tf_	K_DM_	Mg_ww_	Mg_Tf_	Mg_DM_
	**T_1_**										
**TAG**	NS	NS	NS	NS	NS	NS	NS	NS	0.44 *P* = 0.04	0.51 *P* = 0.01	NS
**DM**		NS	0.90 *P*<0.0001	0.86 *P*<0.0001	NS	0.80 P<0.0001	0.71 *P* = 0.0002	NS	0.82 *P*<0.0001	0.80 *P*<0.0001	NS
**DMI**	-0.47 *P* = 0.004		-0.44 *P* = 0.009	-0.57 *P* = 0.005	NS	-0.50 *P* = 0.002	-0.54 *P* = 0.007	NS	-0.44 *P* = 0.009	-0.50 *P* = 0.01	NS
	**T**_**2**_										
**TAG**	NS	NS	NS	NS	NS	NS	NS	NS	NS	NS	NS
**DM**		NS	0.81 *P*<0.0001	0.81 *P*<0.0001	NS	0.82 *P*<0.0001	0.81 *P*<0.0001	NS	0.77 *P*<0.0001	0.76 *P*<0.0001	NS
**DMI**	NS		NS	NS	NS	NS	NS	NS	NS	NS	NS
	**T**_**3**_										
**TAG**	0.54 *P* = 0.001	NS	NS	NS	-0.68 *P*<0.0001	NS	NS	-0.75 *P*<0.0001	NS	NS	-0.73 *P* = 0.03
**DM**		NS	0.45 *P* = 0.006	0.55 *P* = 0.0007	-0.40 *P* = 0.02	NS	0.38 *P* = 0.03	-0.53 *P* = 0.001	NS	0.38 *P* = 0.003	-0.51 *P* = 0.002
**DMI**	NS		NS	NS	NS	NS	NS	NS	NS	NS	NS
	**T**_**4**_										
**TAG**	0.90 *P*<0.0001	-0.65 *P*<0.0001	-0.71 *P*<0.0001	NS	-0.92 *P*<0.0001	NS	0.45 *P* = 0.01	-0.79 *P*<0.0001	-0.50 *P* = 0.004	NS	-0.89 *P*<0.0001
**DM**		NS	-0.51 P = 0.004	NS	-0.90 *P*<0.0001	NS	0.53 *P* = 0.002	0.74 *P*<0.006	NS	0.41 *P* = 0.03	-0.87 *P*<0.0001
**DMI**	-0.52 *P* = 0.0029		0.50 *P* = 0.005	NS	0.57 *P* = 0.0011	NS	-0.43 *P* = 0.02	0.43 *P* = 0.02	0.39 *P* = 0.03	NS	0.58 *P* = 0.0007
	**T**_**5**_										
**TAG**	0.67 P<0.02	NS	NS	NS	-0.70 *P* = 0.02	NS	NS	-0.54 *P* = 0.03	NS	NS	-0.79 *P* = 0.004
**DM**		NS	NS	NS	NS	NS	NS	-0.54 *P* = 0.03	NS	NS	NS
**DMI**	-0.52 *P* = 0.04		NS	NS	NS	NS	NS	NS	NS	NS	NS

Spearman correlation coefficients (r and *P*-values) of liver tissue phosphorus, potassium and magnesium content in liver tissue wet weight (P_WW_, K_WW_ and Mg_WW_ respectively), in fat-free wet weight (P_Tf_, K_Tf_ and Mg_Tf_ respectively) and dry matter (P_DM_, K_DM_ and Mg_DM_ respectively) stratified by sampling times T_1_ (end of acclimation), T_2_ (after two weeks of dietary P deprivation), T_3_ within the first week of lactation, T_4_ (end of deprivation) and T_5_ (only for cows of replicate 1, after two weeks of repletion)

P_DM_, K_DM_ and Mg_DM_ determined at T_3_, T_4_ and T_5_ showed strong negative associations with liver TAG and to a lesser degree with liver tissue DM. The associations were strongest at T_4_ when the highest liver TAG values were recorded and were of similar degree for P, K and Mg (Table 7). Associations were also identified between liver electrolytes and DMI but were considerably weaker than the associations of liver P, K and Mg with liver TAG and liver DM (Table 7).

#### Associations between plasma and liver biochemical parameters

The results of the correlation analysis of plasma biochemical- with liver biochemical parameters are summarized in Table 8. Positive associations with plasma [Pi] were found for liver P_DM_ but also Mg_DM_ at T_2_ and T_4_. Associations of opposite sign with plasma [Pi] were identified for liver P_DM_, K_DM_ and Mg_DM_ at T_5_, which were accompanied by an even stronger positive correlation of plasma [Pi] with liver TAG (Table 8). The plasma GLDH activity at T_4_ was strongly and positively associated with liver TAG, and showed weaker negative associations with liver P_DM_, K_DM_ and Mg_DM_ as well as a positive association with liver DM (Table 8). Similarly TBil at T_4_ was positively associated with the liver TAG and to a lesser degree with liver DM, while negative associations were identified with P_DM_, K_DM_, Mg_DM_ and DMI (Table 8).

**Table 8:** Results of the correlation analysis of dry matte intake (DMI), and parameters of the liver biochemical analysis with blood biochemical parameters.

	DMI	DM	P_DM_	K_DM_	Mg_DM_	TAG
**T**_**1**_						
**Pi**	0.49;*P* = 0.003	NS	NS	NS	NS	NS
**AST**	NS	NS	NS	NS	NS	NS
**GLDH**	NS	NS	NS	NS	NS	0.46;*P* = 0.003
**γGT**	NS	NS	NS	NS	NS	0.52;*P* = 0.02
**TB il**	NS	NS	NS	NS	NS	NS
**NEFA**	NS	NS	NS	NS	NS	NS
**BHBA**	NS	NS	NS	NS	NS	NS
**T**_**2**_						
**Pi**	NS	NS	0.68;*P*<0.0001	0.43;*P* = 0.009	0.52;*P* = 0.001	NS
**AST**	NS	NS	NS	NS	NS	NS
**GLDH**	NS	NS	NS	NS	NS	NS
**γGT**	-0.33;*P* = 0.05	0.40;*P* = 0.02	NS	NS	NS	NS
**TBil**	NS	0.40;*P* = 0.02	NS	NS	NS	NS
**NEFA**	NS	NS	NS	NS	NS	NS
**BHBA**	NS	NS	NS	NS	NS	NS
**T**_**3**_						
**Pi**	NS	NS	NS	NS	NS	NS
**AST**	NS	NS	NS	NS	NS	NS
**GLDH**	NS	NS	NS	NS	NS	NS
**γGT**	NS	NS	NS	NS	0.36;*P* = 0.03	NS
**TBil**	NS	NS	NS	NS	NS	0.48;*P* = 0.004
**NEFA**	NS	0.47;*P* = 0.003	NS	MS	-0.37;*P* = 0.03	0.62;*P*<0.0001
**BHBA**	NS	NS	NS	-0.38;*P* = 0.02	-0.36;*P* = 0.03	0.43;*P* = 0.01
**T**_**4**_						
**Pi**	0.59;*P* = 0.0004	NS	0.36;*P* = 0.05	NS	0.40;*P* = 0.03	-0.37;*P* = 0.04
**AST**	NS	NS	NS	NS	NS	NS
**GLDH**	NS	0.37;*P* = 0.04	-0.44;*P* = 0.01	-0.41;*P* = 0.02	-0.38;*P* = 0.04	0.49;*P* = 0.006
**γGT**	NS	NS	NS	NS	NS	NS
**TBil**	-0.44;P = 0.01	0.51;*P* = 0.004	-0.57;*P* = 0.001	-0.59;*P* = 0.0007	-0.61;*P* = 0.0003	0.54;*P* = 0.002
**NEFA**	-0.41;*P* = 0.002	0.43;*P* = 0.02	-0.58;*P* = 0.0008	-0.69;*P*<0.0001	-0.64;*P* = 0.0002	0.51;*P* = 0.004
**BHBA**	NS	0.40;*P* = 0.02	-0.39;*P* = 0.03	NS	-0.43;*P* = 0.02	0.45;*P* = 0.01
**T**_**5**_						
**Pi**	NS	NS	-0.60;*P* = 0.01	-0.62;*P* = 0.01	-0.59;*P* = 0.01	0.77;*P* = 0.005
**AST**	NS	NS	NS	NS	NS	NS
**GLDH**	NS	NS	NS	NS	NS	NS
**γGT**	NS	NS	NS	NS	NS	NS
**TBil**	NS	NS	NS	NS	NS	NS
**NEFA**	-0.51;*P* = 0.04	0.53;*P* = 0.03	NS	NS	NS	NS
**BHBA**	-0.55;*P* = 0.03	NS	NS	NS	NS	0.66;*P* = 0.03

Spearman correlation coefficients (r and *P*) for dry matter intake (DMI), liver tissue dry matter (DM), liver tissue content on dry matter basis of P (P_DM_), potassium (K_DM_) and magnesium (Mg_DM_) as well as liver tiacylglycerol (TAG) with the plasma inorganic phosphate- (Pi), total bilirubin- (TBil), non-esterified fatty acid- (NEFA), betahydroxybutyrate- (BHBA) concentration as well as the activity in plasma of aspartate aminotransferase (AST), glutamate dehydrogenase (GLDH) and gammaglutamyl transferase (γGT) stratified by sampling times T_1_ (end of acclimation), T_2_ (after two weeks of dietary P deprivation), T_3_ within the first week of lactation, T_4_ (end of deprivation) and T_5_ (only for cows of replicate 1, after two weeks of repletion)

#### Relative abundance of specific mRNA in liver tissue

The relative abundances of mRNA in liver tissue stratified by sampling time, treatment and replicate are presented in Tables 9 and 10. A treatment effect was only identified for ACC with slightly higher values for LP than C (Tables 9 and 10). Time effects were found for all enzymes with exception of PFKL and ACC, with higher values after than before parturition. Treatment x time interaction effects were identified for cPEPCK and PFKL, both with higher values for LP than C at T_3_. Differences between replicates were again significant in all instances with exception of ACC, with higher values in replicate 1 than replicate 2 with exception of Alb. A replicate x treatment interaction effect was not observed (Tables 9 and 10).

**Table 9:** Results of the analysis of variance of relative abundance of specific liver mRNA.

Item	Effects				
	Tx	Time	Replicate	Tx • Time	Tx • Replicate
**cPEPCK**	N.S	<0,0001	0,0005	0.0089	N.S
**PC**	N.S	<0,0001	0.0101	N.S	N.S
**PFKL**	N.S	NS	0.0225	0.0385	N.S
**Alb**	N.S	0.0006	0.0482	N.S	N.S
**ACC**	0.0330	N.S	N.S	N.S	N.S
**SREBF1**	N.S	<0,0001	0,0127	N.S	N.S

Studied enzymes were cytosolic phosphoenolpyruvate carboxykinase (cPEPCK), pyruvate carboxylase (PC), liver pyruvate kinase (PKL), liver phosphofructokinase (PFKL), albumin (Alb), acetyl CoA carboxylase (ACC) and sterol regulatory element binding factor 1 (SREBF1); Tx = Treatment.

**Table 10:** Results of the analysis of variance of the relative abundance of specific liver mRNA stratified by sampling time, treatment and replicate.

Item	Tx	Sampling time					Treatment	Replicate	
		T_1_	T_2_	T_3_	T_4_	T_5_		1	2
**cPEPCK**^**1**^	LP	24.19 [23.98–24.95]	24.12 [23.24–24.88]	24.28* [23.54–25.86}	23.63 [23.00–24.22}	23.97 [23.76–24.36]	24.08 [23.59–24.88]	24.19^a^ [23.94–24.93]	23.96^b^ [23.00–28.74]
	C	25.08^a^ [24-43-25.29]	24.54^a^ [24.01–25.10]	23.54^b^ [23.16–24.41]	23.39^b^ [23.11–23.71]	23.75^b^ [23.40–24.24]	24.06 [23.44–25.05]	24.38^a^ [23.55–25.06]	23.89^b^ [23.17–24.73]
**PC**	LP	28.44^a^ [26.46–30.43]	27.61^ab^ [25.62-29-59]	26.92^b^ [24.93–28.90]	27.29^ab^ [25.29–29.29]	28.38^ab^ [26.38–30.38]	27.67 [25.70–29.64]	28.25^a^ [26.28–30.22]	27.42^b^ [25.44–29.39]
	C	29.04^a^ [27.06–31.02]	28.77^a^ [26.7–30.76]	26.79^b^ [24.81–28.77]	27.67^ab^ [25.69–29.54]	28.31^ab^ [26.32–30.31]	28.31 [26.34–30.29]	28.31 [26.34–30.29]	27.93 [25.95–29.90]
**PFKL**^**1**^	LP	29.26 [28.96–29.58]	29.14 [28.57–29.48]	29.62* [29.02–30.74]	29.16 [28.91–29.75}	30.18 [29.23–30.29]	29.27 [28.91–29.80]	29.44 [29.10–29.95]	29.06 [28.74–29.61]
	C	29.44 [28.99–29.78]	29.29 [28.92–29.85]	28.95 [28.71–29.10]	29.20 [29.06–29.85]	29.66 [29.45–29.93]	29.26 [28.92–29.78]	29.45 [29.06–29.90]	29.05 [28.86–29.56]
**Alb**	LP	17.46^a^ [15.47–19.46]	17.53^a^ [15.54–19.53]	19.18^b^ [17.18–21.17]	18.60^ab^ [16.60–20.60]	18.53^ab^ [16.50–20.55]	18.25 [16.27–20.23]	17.70^a^ [15.72–19.68]	18.41^b^ [16.38–20.43]
	C	17.96 [19.97–19.96]	17.90 [15.91–19.90]	18.49 [16.49–20.48]	18.49 [16.49–20.48]	18,22 [16.21–20.24]	18.23 [16.25–20.21]	18.16 [16.18–20.13]	18.75 [16.76–20.74]
**ACC**	LP	31.19 [29.21–33.17]	30.76 [28.78–32.74]	31.77 [29.79–33.75]	30.90 [28.92–32.89]	31.05 [29.05–33.04]	31.12* [29.15–33.09]	30.97 [29.00–32.95]	31.05 [29.05–33.04]
	C	30.69 [28.71–32.67]	30.55 [28.57–32.53]	31.12 [29.14–33.10]	30.48 [28.50–32.46]	30.34 [28.35–32.33]	30.62 [28.65–32.59]	30.76 [28.79–32.73]	31.26 [29.28–33.24]
**SREBF1**	LP	29.85^ab^ [27.88–31.83]	29.58^a^* [27.60–31.56]	31.48^b^ [29.50–33.46]	30.13^a^ [28.15–32.11]	30.34^ab^ [28.35–32.33]	30.27 [28.30–32.25]	30.55 [28.58–32.52]	29.99 [28.02–31.97]
	C	30.20 [28.22–32.18]	30.48 [28.50–32.46]	30.90 [28.93–32.88]	30.69 [28.71–32.67]	30.69 [28.71–32.67]	30.62 [28.65–32.59]	30.76 [28.79–32.73]	30.41 [28.44–32.38]

Least square means and 95% confidence interval or median and interquartile range of cytosolic phosphoenolpyruvate carboxykinase (cPEPCK), pyruvate carboxylase (PC), liver pyruvate kinase (PKL), liver phosphofructokinase (PFKL), albumin (Alb), acetyl CoA carboxylase (ACC) and sterol regulatory element binding factor 1 (SREBF1) stratified by sampling time (T_1_: end of acclimation, T_2_: after two weeks of dietary P deprivation, T_3:_: within the first week of lactation, T_4_: end of deprivation, and T_5_: only for cows of replicate 1, after two weeks of repletion), by treatment (LP: low phosphorus, C: control) and replicate. Values with different superscript letters within one line differ significantly (*P*<0.05, Bonferroni corrected); values with asterisk (*) differ between treatments within one row.

^1:^ Results are presented as median and interquartile range.

#### Associations between relative mRNA abundance of specific liver enzymes and liver biochemical parameters

The results of the correlation analyses between liver biochemical parameters and relative abundance of specific liver mRNA are summarized in Table 11. The relative abundance of SREBF1 was positively associated with the liver tissue content of P, Mg and K when expressed on a DM- or WW basis at T_1_ and T_2_ (Table 11). At T_1_ SREBF1 also showed a positive association with liver DM. At T_2_ and T_3_ the relative abundance of cPEPCK was positively associated with liver P, K and Mg primarily when expressed on a WW basis. Alb was negatively associated with liver DM at T_2_ and T_3_, as well as with P_WW_ and K_WW_ at T_1_ and T_2_ and was positively associated with K_DM_ and Mg_DM_ at T_3_ (Table 11).

**Table 11:** Results of the correlation analysis of liver tissue biochemical parameters and relative abundance of mRNA of specific lover enzymes.

Item	cPEPCK	PC		PFKL	Albumin	ACC	SREBF1
**T**_**1**_							
**P**_**DM**_	N.S	N.S		N.S	N.S	N.S	N.S
**P**_**WW**_	N.S	N.S		N.S	-0.43;*P* = 0.01	N.S	0.47;*P* = 0.005
**K**_**DM**_	N.S	N.S		N.S	N.S	N.S	N.S
**K**_**WW**_	N.S	N.S		N.S	N.S	N.S	0.43;*P* = 0.01
**Mg**_**DM**_	N.S	N.S		N.S	N.S	N.S	N.S
**Mg**_**WW**_	N.S	N.S		N.S	N.S	N.S	0.49;*P* = 0.003
**DM**	N.S	N.S		N.S	N.S	N.S	0.47;*P* = 0.004
**TAG**	N.S	N.S		N.S	N.S	N.S	N.S
**T**_**2**_							
**P**_**DM**_	N.S	N.S		N.S	N.S	N.S	0.47;*P* = 0.003
**P**_**WW**_	0.35;*P* = 0.04	N.S		0.34;*P* = 0.04	N.S	N.S	0.50;*P* = 0.003
**K**_**DM**_	N.S	N.S		N.S	N.S	-0.41;*P* = 0.01	N.S
**K**_**WW**_	0.36;*P* = 0.04	N.S		N.S	-0.40;*P* = 0.02	N.S	0.38;*P* = 0.02
**Mg**_**DM**_	N.S	N.S		N.S	N.S	N.S	0.38;*P* = 0.02
**Mg**_**WW**_	N.S	N.S		N.S	N.S	N.S	0.45;*P* = 0.007
**DM**	0.39;*P* = 0.02	0.41;*P* = 0.01		N.S	-0.36;*P* = 0.03	N.S	N.S
**TAG**	N.S	N.S		N.S	N.S	N.S	N.S
**T**_**3**_							
**P**_**DM**_	N.S		N.S	N.S	N.S	N.S	N.S
**P**_**WW**_	N.S		N.S	N.S	N.S	N.S	N.S
**K**_**DM**_	N.S		N.S	N.S	0.42;*P* = 0.01	N.S	N.S
**K**_**WW**_	0.49;*P* = 0.003		N.S	N.S	N.S	N.S	N.S
**Mg**_**DM**_	0.39;*P* = 0.02		N.S	N.S	0.42;*P* = 0.01	N.S	N.S
**Mg**_**WW**_	0.42;*P* = 0.01		N.S	N.S	N.S	N.S	N.S
**DM**	N.S		N.S	N.S	-0.37;*P* = 0.03	N.S	N.S
**TAG**	N.S		N.S	N.S	N.S	N.S	N.S
**T**_**4**_ **and T**_**5**_							
**P**_**DM**_	N.S	N.S		N.S	N.S	N.S	N.S
**P**_**WW**_	N.S	N.S		N.S	N.S	N.S	N.S
**K**_**DM**_	N.S	N.S		N.S	N.S	N.S	N.S
**K**_**WW**_	N.S	N.S		N.S	N.S	N.S	N.S
**Mg**_**DM**_	N.S	N.S		N.S	N.S	N.S	N.S
**Mg**_**WW**_	N.S	N.S		N.S	N.S	N.S	N.S
**DM**	N.S	N.S		N.S	N.S	N.S	N.S
**TAG**	N.S	N.S		N.S	N.S	N.S	N.S

Spearman correlation coefficients (r and *P*) liver tissue of phosphorus, potassium and magnesium on adry matter basis (P_DM_, K_DM_ and Mg_DM_ respectively) and wet weight basis (P_WW_, K_WW_ and Mg_WW_ respectively), liver tissue dry matter content (DM) and liver tissue triacylglycerol content with the relative abundance of mRNA in liver tissue for the synthesis of cytosolic phosphoenolpyruvate carboxykinase (cPEPCK), pyruvate carboxylase (PC), liver pyruvate kinase (PKL), liver phosphofructokinase (PFKL), albumin (Alb), acetyl CoA carboxylase (ACC) and sterol regulatory element binding factor 1 (SREBF1) stratified by sampling time (T_1_: end of acclimation, T_2_: after two weeks of dietary P deprivation, T_3:_: within the first week of lactation, T_4_: end of deprivation, and T_5_: only for cows of replicate 1, after two weeks of repletion).

None of the studied liver enzymes was associated with plasma [Pi] at any of the sampling times. PC was negatively associated plasma NEFA at T_2_ (r = -0.45; *P* = 0.006), T_3_ (r = -0.36; *P* = 0.03) and T_5_ (r = -0.54; *P* = 0.03) and with plasma [BHBA] at T_3_ and T_5_ (r = -0.36; *P* = 0.03 and r = -0.49; *P* = 0.05). PFKL was negatively associated with plasma NEFA (r = -0.45; *P* = 0.006) and plasma GLDH (r = -0.40; *P* = 0.02) at T_2_ as well as with plasma TBil (r = -0.42; *P* = 0.02) and plasma NEFA (r = -0.49; *P* = 0.005) at T_4_. cPEPCK showed a significant negative correlation with plasma BHBA at T_3_ (r = -0.40; *P* = 0.01).

## Discussion

The objective of the two studies presented here was to determine how hepatocytes and liver function of dairy cows respond to P deprivation and hypophosphatemia. The focus of these studies was on effects on biochemical composition of liver tissue, alteration of metabolic activity and signs of liver injury occurring with P deprivation.

### In-vitro study

A stable positive glucose balance, low LDH activity in culture medium, stable urea synthesis and low transcription rates of NFkBIα, a marker for programmed cell death in cultured cells confirm that the sandwich culture technique employed here successfully maintained bovine hepatocytes viable and functional over a period of 168h. This is consistent with the results of earlier studies having employed the same culture technique ([28]).

This in-vitro approach allowed identifying a significant effect of the Pi concentration in culture medium on the glucose output of incubated hepatocytes, but not on the transcription rates of key enzymes of the carbohydrate metabolism in these cells. The higher glucose output of cells incubated in LP medium than of cells incubated in HP medium refutes the hypothesis that restricted availability of Pi in the extracellular environment over a period of several days may impair the hepatic carbohydrate metabolism.

Medium without and with high glucose concentration was also included to study the response of hepatocytes in-vitro to treatments presumably presenting important challenges for the carbohydrate metabolism. As expected large differences in the glucose output were identified with a glucose balance of cells incubated in high glucose medium in the range of only 1/10 of the balance of cell incubated without glucose. Comparison of the relative abundance of mRNA of enzymes of the carbohydrate metabolism in contrast only revealed a small effect on the transcription rate of cPEPCK, for which a lower relative abundance of mRNA was measured in cells incubated at high glucose than cells incubated without glucose. cPEPCK is a rate limiting enzyme of the gluconeogenic pathway and changes in cPEPCK activity are highly predictive for hepatic glucose production [29]. These results indicate that a rapid and quantitatively important adjustment of the hepatocytic glucose output can occur in the short term with only small changes of the transcription rates of enzymes of the carbohydrate metabolism. Studying liver metabolites thus seems to be a more sensitive approach to assess the carbohydrate metabolic activity than measuring the transcription rate of rate limiting enzymes of the carbohydrate metabolism.

An effect of P depletion was also identified on the LDH activity in incubation medium at 48h and 72h. Culture medium LDH is used as indicator for hepatocyte vitality, with increased activities being indicative for higher enzyme leakage from presumably injured hepatocytes [30]. Higher LDH activities of LPi than IPi media at 48h and 72h may thus be interpreted as indication of a higher vulnerability of P deprived hepatocytes particularly during the early phase of adaptation to the in-vitro environment. Neither LDH activity in culture medium nor transcription rate of NFkBIα during the second half of the study suggest a sustained negative effect of P deprivation on hepatocyte vitality.

Higher LDH activities were also determined in HG medium than in 0G medium, providing indication for a possible deleterious effect of excessive glucose concentrations on hepatocyte vitality. Upregulation of LDH synthesis has however also been reported to occur in response to hyperglycemia [31]. It is thus conceivable that elevated LDH activity in HG medium is not solely the result of cell injury but also of a metabolic response to hyperglycemia. Similar urea concentrations in media without, with intermediate and with high glucose concentration as well as similar transcription rates of NFkBIα are not suggestive of disturbed hepatocyte function or–injury of cells exposed the a high glucose medium.

Overall the results of the in-vitro study do not support our working hypothesis that restricted availability of P to bovine hepatocytes may impair the hepatic carbohydrate metabolism. A negative effect on hepatocyte vitality of cells at least in vitro cannot be ruled out and may deserve further investigation.

A limitation of this experiment is the relatively narrow time frame during which hepatocytes can be studied that is determined by the time required for cells to acclimate to the in-vitro environment and by the maximum survival time of cultivated hepatocytes. With a Pi concentration of 0.9 mmol/L in culture medium, hepatocytes were exposed to an ambient [Pi] equivalent to what would be considered as moderately hypophosphatemic. Negative effects of lower ambient [Pi] on the metabolic activity of hepatocytes in-vitro cannot be ruled out.

In this artificial medium hepatocytes were isolated from endocrine stimuli upregulating the glucose output as this would be expected to occur in highly productive fresh dairy cows. The hepatocytic capacity to upregulate the gluconeogenic activity depending on the availability of P was not investigated in this study, but is certainly a subject of interest for future studies. It is conceivable that P deprivation may have a hampering effect on the capacity of liver cells to upregulate the glucose output.

### In-vivo study

The plasma Pi concentration-time curve confirms the efficacy of the LP diet used in this study in inducing pronounced and sustained hypophosphatemia in P deprived animals. With mean plasma [Pi] below 0.5 mmol/L from the time of parturition in cows assigned to the LP treatment can be considered to have been severely hypophosphatemic throughout the first weeks of lactation [19].

#### Feed intake

The clinically most apparent symptom presumably attributable to dietary P deprivation was a marked feed intake depression that developed in P deprived cows from the second week of lactation on. Feed intake depression or anorexia is a well-recognized symptom of sustained P-deficiency and hypophosphatemia in cattle and other species including humans [6, 7, 32–34]. Similar results in dairy cows, with feed intake depression occurring in the second week of lactation were reported with slightly less pronounced dietary P-deprivation starting approximately 3 weeks before parturition [32]. In contrast, mid lactating dairy cattle fed a similarly P deficient diet over a course of 4 weeks did neither show feed intake depression nor a drop in milk yield [35]. Similarly more moderate P-deprivation that was initiated in mid lactation and extended throughout the dry period and the following lactation resulted in lower feed intake only after dry-off [33]. It remains thus uncertain whether the critical determinant for occurrence of anorexia from P deprivation is the duration of the P shortage or the proximity to parturition when the onset of lactation can rapidly exacerbate P-depletion of the body.

It is furthermore unclear through which mechanism P-deprivation causes anorexia. Altered metabolic activity of the P deprived rumen microbiome has been incriminated as potential cause, a pathway that however does not explain identical symptoms in monogastric species [36]. Disturbances of the central nervous system in states of severe hypophosphatemia may be worthwhile considering as potential cause for the observed symptoms. Neurologic symptoms such as altered demeanor, disorientation and confusion have been consistently reported in severely hypophosphatemic human patients and were found to rapidly resolve with oral or parenteral P supplementation [6]. In rats experimental P depletion was associated with a significant reduction of neurotransmitters in various regions of the central nervous system. It could however not be clarified whether this effect was directly attributable to P depletion or rather was the consequence of an altered energy metabolism that often is coupled with hypophosphatemia [15].

In the present study correction of P-deprivation by feeding a diet with adequate P content rapidly restored the appetite with a return to normal feed intake as this was reported previously [37]. This effect was preceded by an almost immediate correction of hypophosphatemia after initiating dietary P supplementation. The efficacy of oral supplementation of P salts for the rapid and sustained correction of hypophosphatemia in previously P deprived dairy cows has been documented in several studies [38–40].

Experimental rations used in this study differed in their P content that was supplemented in the rations of treatment C as NaH_2_PO_4_. Diets thus not only differed in their P but also the sodium (Na) content. During the planning of this study the decision was made not to adjust Na in the P deficient diets by adding NaCl in order to avoid potential issues with palatability. The objective instead was to keep the dietary Na content within the advised range for both treatments [24]. The measured dietary Na content of the lactation LP diet of 1.5 g/kg DM was below the targeted content of 1.8 g/kg and thus in a marginal range for lactating cows. Although this Na supply was adequate to cover the requirements for 30 kg of milk production assuming normal feed intake, the combination of marginal Na content with sustained anorexia may have resulted in a negative Na balance in early lactation [41]. Because Na deficiency was also shown to negatively affect voluntary feed intake it is conceivable that anorexia observed in LP cows may have been exacerbated by insufficient Na uptake. An earlier study where a diet with similarly low P content and a Na content of 1.8 g/kg DM was fed to mid lactating dairy cows did neither result in measurable depression of feed intake or milk yield [35].

#### Plasma biochemical parameters

The plasma biochemical analysis at the five sampling times showed the characteristic development of transient NEB commonly occurring in early lactating dairy cows [18]. Plasma [NEFA] reached similar peak values for both treatments in the first week of lactation while plasma [BHBA] peaked at 4 weeks post-partum for both treatments, again a characteristic pattern observed in fresh dairy cows [18, 42]. As expected plasma NEFA and BHBA were strongly and positively associated with liver TAG indicating the development of liver lipid accumulation as the severity of NEB progressed.

Although a higher liver TAG content was observed in LP than in C cows as this was expected based on the important differences in feed intake between treatments, a treatment effect was neither identified for plasma NEFA nor for BHBA. This peculiarity may be attributable to the use of propylene glycol in cows with pronounced ketonuria that was more common in LP than in C cows.

No clear treatment- or treatment x time interaction effect was identified for any of plasma biochemical parameters commonly used in clinical pathology to identify liver cell damage or disturbed liver function. The correlation analysis revealed stronger associations of TBil and GLDH with liver TAG than with P_DM_ inferring that disturbed liver function or liver injury is rather associated with the development of hepatic lipidosis secondary to P depletion rather than with P depletion per se. These results thus do not provide indication for increased vulnerability of liver tissue in P deprived transition cows. The weak treatment- as well as the treatment x replicate interaction effect observed for plasma γGT are difficult to interpret in this context due to the conflicting treatment effects in the first and second replicate.

Similarly the treatment x time interaction effect for TBil with higher values in LP cows in the first week after calving cannot unambiguously be attributed to a hepatic cause. It is conceivable that the slightly more elevated TBil values at T_3_ in LP cows are the result of early stage postparturient hemoglobinuria. Although neither clinical nor laboratory diagnostic parameters were suggestive of intravascular hemolysis at T_3_ in any of the LP cows that developed postparturient hemoglobinuria one to two weeks later, it is conceivable that hemolysis and the release of hemoglobin at a subclinical level may have resulted in mild hyperbilirubinemia that thus would have been of prehepatic origin. This assumption is supported by the fact that the observed treatment x time interaction effect became insignificant when TBil values obtained at T_3_ from the 3 LP cows that later developed postparturient hemoglobinuria were excluded from the analysis.

Positive associations of plasma [Pi] with the DMI observed at several time points corroborate the current understanding that feed intake is an important determinant of the extracellular P balance [19]. The opposite causative relation however supports the observation that P deprivation is associated with feed intake depression. Correlations of the plasma [Pi] with liver P_DM_, were identified at several time points but seem to be attributable to collinearity with liver TAG, liver DM and DMI rather than to a P-specific effect. As has been suggested previously the plasma [Pi] must be considered to be poorly suited to assess the liver P content [26].

IGF-1 was studied in experimental cows during late gestation to identify a possible link between this hormone of hepatic origin and the P homeostasis. IGF-1 is thought to contribute to the regulation of the P homeostasis through its proliferative effect on osteoblasts as well as through its association with fibroblast growth factor-23 (FGF23), an endocrine substance recognized as a key regulator of the P homeostasis [43, 44]. Dietary P deprivation in dairy cows results in pronounced osteoclastic activity with bone mobilization [20, 35]. The mechanism through which bone mobilization is initiated in states of hypophosphatemia is not yet understood. Since IGF-1 is associated with enhanced bone mineralization and upregulation of FGF23 it is assumed that in case IGF-1 is contributing to the regulation of the P homeostasis it would be downregulated in states of hypophosphatemia. Although the correlation- and regression analyses indicate a positive association between plasma [Pi] and IGF-1 neither a significant treatment nor treatment x time interaction effect were identified despite of pronounced declines in plasma [Pi] in LP cows between T_1_ and T_2_ and important differences in plasma [Pi] between LP and C at T_2_. This infers that the plasma [Pi] is not a major determinant of the hepatic IGF-1 synthesis at least in dairy cows in late gestation.

#### Liver tissue biochemical analysis

The most prominent treatment effect on liver tissue was the more pronounced TAG accumulation in liver tissue in LP cows at the sampling times T_4_ and T_5_ that was more than double the amount determined for treatment C. This effect coincides with the considerably lower feed intake in LP than in C cows after calving and thus is presumably attributable to an exacerbated NEB in P deprived cows developing anorexia.

The increase in liver TAG in P deprived cows was associated with an increase in liver DM i.e. a decrease in tissue water content, an association that also reflects in the strong positive correlation between liver TAG and liver DM at the sampling times T_3_ to T_5_. Negative associations between liver lipid and tissue water content have been reported earlier in cattle and other species and are thought to be driven by a compensatory mechanism regulating the cell volume that is critical to maintain liver cell and organ function [26, 45, 46]. A reduction of the cell water volume in response the cell lipid accumulation results in a reduced volume of distribution of water soluble intracellular electrolytes and other solutes. Consequently a dehydrated cell must lower the intracellular electrolyte content to correct the osmotic disequilibrium resulting from cell water volume reduction [26]. To differentiate between changes in liver electrolyte content attributable to cytosol volume changes from those attributable to other mechanism such as dietary deprivation, liver Pi, K and Mg contents, all predominantly intracellular electrolytes, were expressed on a DM-, a wet weight and a TAG-free wet weight basis. A change in tissue electrolyte content expressed on DM basis can be the result of altered tissue electrolyte content with constant cell water volume, an altered tissue water volume with constant electrolyte content or altered electrolyte content together with altered tissue water volume. Expression of the liver electrolyte content on a wet weight basis takes into consideration a concomitant change in tissue water thereby reducing the effect attributable to alteration of the tissue water content. Altered liver electrolyte contents expressed on wet weight basis are thus more indicative of a change in liver electrolyte content rather than cell volume changes. Because the tissue water content is strongly and negatively correlated with the highly variable liver TAG content we attempted to improve the estimation of cytosol volume by subtracting the amount of TAG from the tissue mass.

All three liver electrolytes showed similar concentration—time curves when expressed on a DM basis, and treatment effects were significant for all of them. Treatment effects were still apparent when expressing P on a TAG-free wet weight basis, but vanished for K_TF_ and Mg_TF_. From these results it can be concluded that the observed reduction in liver tissue K and Mg content in LP cows is primarily attributable to liver TAG accumulation and the ensuing reduction of cytosol volume while for Pi the restricted dietary P supply in LP cows also contributed to the reduction in liver tissue P content. Although the liver P content was significantly lower in LP than C cows after two weeks of P deprivation already, values were restored at calving suggesting that the liver is able to maintain the intracellular P balance for weeks despite of pronounced hypophosphatemia during late gestation. It is with the onset of lactation that the regulatory mechanisms of the intracellular P homeostasis seem to become overwhelmed as is reflected in the continuous decline of the liver P content throughout the lactation period. It is remarkable that the liver P content of LP cows continued to decline during the repletion phase of the study despite of the increased oral supply and the associated rebound of the plasma [Pi], suggesting that not only P depletion but also P repletion is slow to occur. Part of this slow response of the liver to P supplementation might be due to the higher liver TAG content and the slow clearance of these lipids from the hepatocyte. The slow response of the liver and the almost immediate response of plasma [Pi] to P supplementation likely resulted in the seemingly paradoxical inversion of observed correlations between plasma [Pi] and P_DM_ and liver TAG at T_5_ compared to the previous sampling time points.

Significant replicate effects were observed for liver TAG and liver DM with higher TAG and higher DM in replicate 1 than in replicate 2. These replicate effects evidently also reflect in the electrolyte contents expressed on WW- and TAG-free basis since these were derived from liver tissue DM and TAG. Replicate effects were not apparent for liver electrolyte contents expressed on DM basis. These results indicate that NEB may have been more pronounced in cows of replicate 1 than replicate 2. Because differences between replicates were apparent for both treatments with deem differences in quality of ration ingredients used in both treatments to be the probable cause of this effect. Differences in severity of NEB between replicates would furthermore explain the lower IGF-1 levels in replicate 1 than replicate 2 [47].

#### Liver metabolic activity

In view of the lack of marked effects of P deprivation on the transcription rate of liver enzymes of the gluconeogenic and glycolytic pathway as determined in the in-vitro study presented here, the range of studied liver enzymes was expanded in this in-vivo study in order to broaden the variety of liver metabolic pathways under investigation. The hepatic carbohydrate metabolism was again studied on the example of the relative abundance of hepatic mRNA of cPEPCK, PC and PFKL. The slightly higher relative abundance of cPEPCK in LP at the first week of lactation does again not support the hypothesis that P deprivation in late gestation may hamper the gluconeogenic activity of the liver. Similarly PC, the enzyme catalyzing the conversion of pyruvate to oxaloacetate and thereby fine tuning the regulation of the entry of endogenous glucose precursors into gluconeogenesis was not affected by dietary P deprivation. In the present study cPEPCK transcription rates were at their nadir at T_4_ for both treatments when the liver TAG contents peaked. The difference in liver TAG between treatments at T_4_ did however not result in measurable differences in relative abundance of cPEPCK mRNA. The glycolytic activity was again assessed by studying PFKL, a rate-limiting enzyme of the glycolytic pathway. PFKL catalyzes the first irreversible reaction of this pathway that is the conversion of fructose-6-phosphate to fructose-1-6-biphosphate. This reaction is upregulated with high ambient adenosine monophosphate concentration and downregulated in an environment with high ATP content. PFKL thus up- or down regulates the cellular glycolytic activity as the cellular energy density lowers or rises respectively. Differences between treatments were again only observed at T_3_ with slightly higher values in LP cows, again suggesting that P deprivation did not hamper the glycolytic activity of the liver of P deprived animals.

The relative abundance in liver mRNA of sterol regulatory element-binding transcription factor 1 (SREBF1) and acetyl-CoA carboxylase (ACC) were studied to assess the hepatic lipid metabolism. SREBF1 plays a pivotal role in the regulation of the de-novo cholesterol biosynthesis; its regulation strongly depends on blood cholesterol levels and feed intake. Upregulation of this enzyme as it was predominantly observed in LP cows has been reported to occur in healthy dairy cows after calving [48]. ACC, an enzyme of the fatty acid biosynthesis catalyzing the conversion from acetyl CoA to malonyl-CoA was the only enzyme for which a weak but significant treatment effect was identified [49]. Slightly higher values in LP than in C cows are indicative of higher hepatic de-novo lipid synthesis in states of P-deprivation. Dietary P deprivation did thus not seem to impair the liver lipid metabolism in transition dairy cows.

Finally the protein metabolism was studied on the example of the transcription rate of the albumin gene. An upregulation of the albumin synthesis after parturition was most apparent in LP cows but showed a similar trend in cows of treatment C, which again does not suggest a disruption of the protein anabolic activity of the P deprived liver.

The positive associations of the relative abundance of mRNA with liver DM and to a lesser extent with liver electrolytes when expressed on a wet weight basis suggest a concentration of mRNA that increases as tissue water decreases. Such associations were determined for all enzymes with exception of Alb and ACC and only at the sampling times T_1_ to T_3_ when liver TAG concentrations were still low. For none of the enzyme transcription rates an association with the liver TAG content was observed at any of the five sampling times. This implies that the levels of liver lipid accumulation observed in the present study did not markedly affect enzyme transcription rates. Remarkably for Alb a negative association was determined with liver DM and some of the electrolytes when expressed on a wet weight basis. Positive associations with liver electrolytes when expressed on a DM basis only observed at the sampling times T_1_ to T_3_ suggest that the transcription rate of the genes encoding albumin is downregulated as the liver cell dehydrates.

Considering the differences in liver tissue DM between replicates, with higher liver DM in replicate 1 than replicate 2 and the higher relative abundances in mRNA of all studied enzymes except Alb and ACC in replicate 1 than replicate 2 it is conceivable that the difference in liver DM in both replicates is again the cause for the observed replicate effect for cPEPCK, PC, PFKL and SREBF1. For albumin mRNA transcription rates were lower in replicate 1 than replicate 2 which is in agreement with the negative correlations between Alb and liver DM mentioned above.

#### Animal health

Neither clinically apparent signs of muscle weakness nor cases of recumbency were observed in P deprived animals. Effects of dietary P deprivation on calcium homeostasis around parturition and on the function and biochemical composition of muscle tissue in these animals have been reported elsewhere [20, 22].

Another remarkable clinical outcome presumably attributable to dietary P deprivation was the occurrence of postparturient hemoglobinuria in 3 out of 18 cows of treatment LP but in none of the cows of treatment C. Postparturient hemoglobinuria is a condition characterized by intravascular hemolysis occurring in early lactation that has been empirically associated with P deficiency in cattle. The mechanism through which P deficiency may induce erythrolysis remains uncertain, but intracellular P depletion and ensuing intracellular ATP deficiency that are thought to result in increased osmotic fragility of erythrocytes has been proposed [50]. As it was the case in the present study postparturient hemoglobinuria was found to only occur in a small subset of P deficient animals which lead to the assumption that P deprivation may be a contributing factor but not the sole cause of this condition [51, 52].

#### Limitations

A major difficulty of the studies presented here was to differentiate between direct and indirect effects of P deprivation at the animal-, organ- and cell level. In particular anorexia that developed at the onset of lactation in P deprived cows not only exacerbated the NEB but certainly also contributed to exacerbation of P deprivation and may have caused or contributed to deficiencies of other nutrients potentially confounding the results of this study. The absence of a negative effect of P deprivation that was determined in several instances in this study is however unlikely to be confounded and therefore does not lose validity.

The focus of this experiment was on identifying primary effects of P deficiency which is why it was attempted to moderate the severity of the NEB by supplementing propylene glycol to cows with profound ketonuria. This procedure is likely to have moderated the consequences of NEB for instance in the form of liver TAG accumulation, and may therefore have prevented measurable effects on liver function or liver composition. These effects however would have to be classified as secondary effects of P deficiency attributable to anorexia rather than direct effects of P deprivation.

#### Conclusion

The results of these studies confirm that pronounced hypophosphatemia can be induced by dietary P deprivation in transition cows. Despite of profound hypophosphatemia liver P depletion was only mild and slow to occur. Most prominent effect of sustained P deprivation is the development of anorexia that occurred after parturition and resulted in pronounced liver TAG accumulation. Hepatic lipidosis but not hypophosphatemia or P depletion of liver tissue was associated with a response of plasma biochemical parameters indicating disturbed liver function and exacerbation of the NEB. Hypophosphatemia and P deprivation could not be associated with alterations of the carbohydrate or liver lipid metabolism in P deprived cows or in in-vitro cultivated hepatocytes. From these results we conclude that the most relevant effect of dietary P deprivation in transition cows is feed intake depression causing or exacerbating the development of hepatic lipidosis.

## Supporting information

S1 Table (a) Additives and composition of the daily cell culture medium based on a William’s E Medium. (b) Composition of Williams E Medium.
(DOCX)

S2 Table Primer sequences for RT-qPCR. (included in main document as well to maintain order of references).
(DOCX)

S3 Table Ingredients and composition of experimental rations. (DOCX)

S1 Dataset Raw data of in-vivo and in-vivo study. Exced file containing data from the in-vivo study (data in-vivo) as well as from the in-vitro study obtained in medium (data in-vivo medium) and in bovine primary hepatocytes (in-vitro mRNA). Spreadsheet lableled “legends” provides legends for all tables.
(XLSX)
