# Supplementary material for: Liver phosphorus content and liver function in states of phosphorus deficiency in transition dairy cows
Source: PLoS One. 2019 Jul 22;14(7):e0219546. doi: 10.1371/journal.pone.0219546 (PMC6645509; doi:10.1371/journal.pone.0219546)
Supplement: S1 Table — (a) Additives and composition of the daily cell culture medium based on a William’s E Medium. (b) Composition of Williams E Medium. (DOCX) [file pone.0219546.s001.docx]

S1 Table: (a) Additives and composition of the daily cell culture medium based on a William’s E Medium

|  | **Content** | **Concentration** |
| --- | --- | --- |
| Medium Additives | Penicillin/ Streptomycin | 100 U/mL / 0,1 mg/mL |
|  | Gentamycin | 50 µg/mL |
|  | Amphotericin B | 2,5 µg/mL |
|  | L-Glutamine | 4 mM/L |
|  | Dexamethasone | 100 nM/L |
|  | AS | 1 % |
|  | Sodium pyruvate | 1,0 mM/L |
|  | Sodium propionate | 1,25 mM/L |
|  | Glucose | 0, 5 or 10 mM/L |
| Medium basis | Williams E Medium* |  |

**(b) Composition of Williams E Medium**

| **Ingredients** | **Molecular mass** | | **Concentration (mg/L)** | **mM** |  |
| --- | --- | --- | --- | --- | --- |
| **Amino Acids** | | | | | |
| Glycine | | 75.0 | 50.0 | 0.667 | |
| L-Alanine | | 89.0 | 90.0 | 1.011 | |
| L-Arginine | | 174.0 | 50.0 | 0.287 | |
| L-Asparagine-H2O | | 150.0 | 20.0 | 0.133 | |
| L-Aspartic acid | | 133.0 | 30.0 | 0.226 | |
| L-Cysteine | | 121.0 | 40.0 | 0.331 | |
| L-Cystine 2HCl | | 313.0 | 26.07 | 0.083 | |
| L-Glutamic Acid | | 147.0 | 50.0 | 0.340 | |
| L-Histidine | | 155.0 | 15.0 | 0.097 | |
| L-Isoleucine | | 131.0 | 50.0 | 0.382 | |
| L-Leucine | | 131.0 | 75.0 | 0.572 | |
| L-Lysine hydrochloride | | 183.0 | 87.46 | 0.480 | |
| L-Methionine | | 149.0 | 15.0 | 0.101 | |
| L-Phenylalanine | | 165.0 | 25.0 | 0.152 | |
| L-Proline | | 115.0 | 30.0 | 0.261 | |
| L-Serine | | 105.0 | 10.0 | 0.095 | |
| L-Threonine | | 119.0 | 40.0 | 0.33 | |
| L-Tryptophan | | 204.0 | 10.0 | 0.049 | |
| L-Tyrosine disodium salt dihydrate | | 261.0 | 50.65 | 0.194 | |
| L-Valine | | 117.0 | 50.0 | 0.427 | |
| **Vitamins** | | | | | |
| Ascorbic Acid | | 176.0 | 2.0 | 0.011 | |
| Biotin | | 244.0 | 0.5 | 0.002 | |
| Choline chloride | | 140.0 | 1.5 | 0.011 | |
| D-Calcium pantothenate | | 477.0 | 1.0 | 0.002 | |
| Ergocalciferol | | 397.0 | 0.1 | 0.002 | |
| Folic Acid | | 441.0 | 1.0 | 0.002 | |
| Menadione sodium bisulfate | | 276.0 | 0.01 | 0,00003 | |
| Niacinamide | | 122.0 | 1.0 | 0.008 | |
| Pyridoxal hydrochloride | | 204.0 | 1.0 | 0.005 | |
| Riboflavin | | 376.0 | 0.1 | 0.0003 | |
| Thiamine hydrochloride | | 337.0 | 1.0 | 0.003 | |
| Vitamin A (acetate) | | 328.0 | 0.1 | 0.0003 | |
| Vitamin B12 | | 1355.0 | 0.2 | 0,0001 | |
| alpha Tocopherol phos. Na salt | | 554.7 | 0.01 | 0,00001 | |
| i-Inositol | | 180.0 | 2.0 | 0.011 | |
| **Inorganic Salts** | | | | | |
| Calcium chloride (CaCl_2_) (anhyd.) | | 111.0 | 200.0 | 1.80 | |
| Cupric sulfate (CuSO_4_-5H_2_O) | | 250.0 | 1*10^-4^ | 4*10^-7^ | |
| Ferric sulfate (FeSO_4_-7H_2_O) | | 278.0 | 1*10^-4^ | 3.6 *10^-7^ | |
| Magnesium sulfate (MgSO_4_) (anhyd.) | | 120.0 | 97.67 | 0.813 | |
| Manganese sulfate (MnSO_4_-H_2_0) | | 169.0 | 1*10^-4^ | 6 *10^-7^ | |
| Potassium chloride (KCl) | | 75.0 | 400.0 | 5.333 | |
| Sodium bicarbonate (NaHCO_3_) | | 84.0 | 2200.0 | 26.190 | |
| Sodium chloride (NaCl) | | 58.0 | 6800.0 | 117.241 | |
| Sodium phosphate monobasic (NaH_2_PO_4_) anhydrous | | 138.0 | 140.0 | 1.014 | |
| Zinc sulfate (ZnSO_4_-7H_2_O) | | 288.0 | 2*10^-4^ | 7 *10^-7^ | |
| **Other ingredients** | | | | | |
| D-Glucose (Dextrose) | | 180.0 | 2000.0 | 11.111 | |
| Glutathione (reduced) | | 307.0 | 0.05 | 1.6*10^-4^ | |
| Methyl linoleate | | 295.0 | 0.03 | 1*10^-4^ | |
| Sodium pyruvate | | 110.0 | 25.0 | 0.227 | |
