# Supplementary material for: Liver phosphorus content and liver function in states of phosphorus deficiency in transition dairy cows
Source: PLoS One. 2019 Jul 22;14(7):e0219546. doi: 10.1371/journal.pone.0219546 (PMC6645509; doi:10.1371/journal.pone.0219546)
Supplement: S2 Table — (included in main document as well to maintain order of references). (DOCX) [file pone.0219546.s002.docx]

**S2 Table**: **Primer sequences for RT-qPCR.**

| **Gene** | **Primer sequence (5‘ → 3‘)** | | **Am-plikon [bp]** | **Accession Nr.** |
| --- | --- | --- | --- | --- |
| ACC [1] | Forward | CTCTTCCGACAGGTTCAAGC | 248 | AJ_132890 |
|  | Reverse | ACCATCCTGGCAAGTTTCAC |  |  |
| Alb [2] | Forward | GGGGTGTGTTTCGTCGAGAT | 293 | NM_180992.2 |
|  | Reverse | CTCACAGCAGTCAGCCATGT |  |  |
| cPEPCK | Forward | CAACATCCGATTTCCGGGGT | 106 | NM_174737.2 |
|  | Reverse | TGTCAGCTCGATGCCAATCT |  |  |
| HNF4α | Forward | GAATCAACGGCGACATTCGG | 112 | NM_001015557.1 |
|  | Reverse | AAGGCTGGGATGTACTTGGC |  |  |
| NFκBIα | Forward | TGCAGGCCACCAACTACAAT | 175 | NM_001045868.1 |
|  | Reverse | GACACCAGGTCGGGATTCTG |  |  |
| PC [3] | Forward | CCACGAGTTCTCCAACACCT | 108 | NM_177946.4 |
|  | Reverse | TTCTCCTCCAGCTCCTCGTA |  |  |
| PFKL | Forward | GAGGAGCTGTGCATCGTCAT | 169 | NM_001080244.2 |
|  | Reverse | TCTCCACGATGAACACACGG |  |  |
| PKL | Forward | AGGGACCAGAGATACGCACT | 202 | NM_001076176.1 |
|  | Reverse | GAGATGAGCCCGTCGTCAAT |  |  |
| SREBF1 [4] | Forward | CCAGTCGACAGCTCCATT GA | 67 | NM_001113302 |
|  | Reverse | TGCGCGCCACAAGGA |  |  |
| GAPDH [5] | Forward | CAACATCAAGTGGGGTGATG | 202 | NM_001034034.2 |
|  | Reverse | GGCATTGCTGACAATCTTGA |  |  |

ACC = acetyl CoA carboxylase; Alb = albumin; cPEPCK = cytosolic phosphoenolpyruvate carboxykinase; HNF4α = factor hepatocyte nuclear factor 4α; NFκBIα = nuclear factor kappaBlα; PC = pyruvate carboxylase; PFKL = liver phosphofructokinase; PKL = liver pyruvate kinase; SREBF1 = sterol regulatory element binding factor 1; GAPDH = Glyceraldehyde 3-phosphate dehydrogenase.

**References**

1. Mann S, Leal Yepes FA, Wakshlag JJ, Behling-Kelly E, McArt JAA. The effect of different treatments for early-lactation hyperketonemia on liver triglycerides, glycogen, and expression of key metabolic enzymes in dairy cattle. J Dairy Sci. 2018;101(2):1626-37. Epub 2017/12/12. doi: 10.3168/jds.2017-13360. PubMed PMID: 29224880.

2. Panda S, Bisht S, Malakar D, Mohanty AK, Kaushik JK. In vitro culture of functionally active buffalo hepatocytes isolated by using a simplified manual perfusion method. PloS one. 2015;10(3):e0118841. Epub 2015/03/20. doi: 10.1371/journal.pone.0118841. PubMed PMID: 25790478; PubMed Central PMCID: PMCPMC4366187.

3. Zhang Q, Koser SL, Bequette BJ, Donkin SS. Effect of propionate on mRNA expression of key genes for gluconeogenesis in liver of dairy cattle. J Dairy Sci. 2015;98(12):8698-709. Epub 2015/09/28. doi: 10.3168/jds.2015-9590. PubMed PMID: 26409969.

4. Graber M, Kohler S, Kaufmann T, Doherr MG, Bruckmaier RM, van Dorland HA. A field study on characteristics and diversity of gene expression in the liver of dairy cows during the transition period. J Dairy Sci. 2010;93(11):5200-15. Epub 2010/10/23. doi: 10.3168/jds.2010-3265. PubMed PMID: 20965335.

5. Piechotta M, Kedves K, Araujo MG, Hoeflich A, Metzger F, Heppelmann M, et al. Hepatic mRNA expression of acid labile subunit and deiodinase 1 differs between cows selected for high versus low concentrations of insulin-like growth factor 1 in late pregnancy. J Dairy Sci. 2013;96(6):3737-49. Epub 2013/04/24. doi: 10.3168/jds.2012-6341. PubMed PMID: 23608493.
