# Supplementary material for: Liver phosphorus content and liver function in states of phosphorus deficiency in transition dairy cows
Source: PLoS One. 2019 Jul 22;14(7):e0219546. doi: 10.1371/journal.pone.0219546 (PMC6645509; doi:10.1371/journal.pone.0219546)
Supplement: S3 Table — (DOCX) [file pone.0219546.s003.docx]

**S3 Table: Ingredients and composition of experimental rations**

| Compound | Dry cow diet | | | | | Lactating cows diet | |
| --- | --- | --- | --- | --- | --- | --- | --- |
| (% of DM) | C | | | LP | | C | LP |
| DM (% as fed) | | 60.0 | 59.3 | | 58.3 | | 58.2 |
| NEL (MJ/kg DM) | | 5.90 | 5.88 | | 6.48 | | 6.54 |
| DVE^5^  OEB^6^ (g/d) | | 6.1  18.1 | 6.1  17.9 | | 7.9  11.8 | | 8.0  12.0 |
| Starch | | 11.1 | 11.0 | | 15.3 | | 15.4 |
| Ca | | 0.43 | 0.44 | | 0.58 | | 0.57 |
| K | | 1.35 | 1.34 | | 1.41 | | 1.45 |
| P | | 0.28 | 0.15 | | 0.44 | | 0.20 |
| Mg | | 0.35 | 0.35 | | 0.18 | | 0.18 |
| S | | 0.12 | 0.12 | | 0.14 | | 0.14 |
| Na | | 0.43 | 0.18 | | 0.36 | | 0.15 |
| DCAD (mEq/kg DM) | | 109.9 | 105.0 | | 294.6 | | 290.8 |

Results of the feed analyses of dry- and lactating cow rations of the low phosphorus (LP) and control (C) treatments.

^5^ DVE= intestinal digestible protein [54]

^6^ OEB = degraded protein balance [54]
